# A variant at 9p21.3 functionally implicates *CDKN2B* in paediatric B-cell precursor acute lymphoblastic leukaemia aetiology

**DOI:** 10.1038/ncomms10635

**Published:** 2016-02-12

**Authors:** Eric A. Hungate, Sapana R. Vora, Eric R. Gamazon, Takaya Moriyama, Timothy Best, Imge Hulur, Younghee Lee, Tiffany-Jane Evans, Eva Ellinghaus, Martin Stanulla, Jéremie Rudant, Laurent Orsi, Jacqueline Clavel, Elizabeth Milne, Rodney J. Scott, Ching-Hon Pui, Nancy J. Cox, Mignon L. Loh, Jun J. Yang, Andrew D. Skol, Kenan Onel

**Affiliations:** 1 Department of Pediatrics, University of Chicago, 900 East 57th Street, Room 5140, MC 4060, Chicago, Illinois 60637, USA; 2 Committee on Cancer Biology, University of Chicago, Chicago, Illinois 60637, USA; 3 Department of Medicine, Vanderbilt University Medical Center, Nashville, Tennessee 37232, USA; 4 The Academic Medical Center, University of Amsterdam, Amsterdam 1105 AZ, The Netherlands; 5 Department of Pharmaceutical Sciences, St Jude Children's Research Hospital, Memphis, Tennessee 38105, USA; 6 Committee on Genetics, Genomics and Systems Biology, University of Chicago, Chicago, Illinois 60637, USA; 7 Faculty of Medicine and Health, Information Based Medicine, Hunter Medical Research Institute, School of Biomedical Sciences and Pharmacy, University of Newcastle, New Lambton, New South Wales 2305, Australia; 8 Institute of Clinical Molecular Biology, Christian-Albrechts University, Kiel 24118, Germany; 9 Department of Pediatrics, University Hospital Schleswig-Holstein, Kiel 24105, Germany; 10 INSERM U1153 Epidemiology and Biostatistics Sorbonne Paris Cité Center (CRESS), Epidemiology of Childhood and Adolescent Cancers Team (EPICEA), Villejuif 94807, France; 11 UMRS-1153, Epidemiology and Biostatistics Sorbonne Paris Cité Center (CRESS), Paris-Descartes University, Paris 75270, France; 12 French National Registry of Childhood Hematopoietic Malignancies (RNHE), Villejuif 94807, France; 13 Telethon Kids Institute, University of Western Australia, Crawley, Western Australia 6872, Australia; 14 Division of Molecular Medicine, Pathology North, John Hunter Hospital, Newcastle, New South Wales 2305, Australia; 15 Department of Oncology, St Jude Children's Research Hospital, Memphis, Tennessee 38105, USA; 16 Department of Pediatrics, University of California–San Francisco, San Francisco, California 94143, USA

## Abstract

Paediatric B-cell precursor acute lymphoblastic leukaemia (BCP-ALL) is the most common cancer of childhood, yet little is known about BCP-ALL predisposition. In this study, in 2,187 cases of European ancestry and 5,543 controls, we discover and replicate a locus indexed by rs77728904 at 9p21.3 associated with BCP-ALL susceptibility (*P*_combined_=3.32 × 10^−15^, OR=1.72) and independent from rs3731217, the previously reported ALL-associated variant in this region. Of correlated SNPs tagged by this locus, only rs662463 is significant in African Americans, suggesting it is a plausible causative variant. Functional analysis shows that rs662463 is a *cis*-eQTL for *CDKN2B*, with the risk allele associated with lower expression, and suggests that rs662463 influences BCP-ALL risk by regulating *CDKN2B* expression through CEBPB signalling. Functional analysis of rs3731217 suggests it is associated with BCP-ALL by acting within a splicing regulatory element determining *CDKN2A* exon 3 usage (*P*=0.01). These findings provide new insights into the critical role of the *CDKN2* locus in BCP-ALL aetiology.

## Abstract (web summary)

A risk variant located at 9p21.3 is associated with cancer risk in pediatric B-cell precursor acute lymphoblastic leukaemia. Here, the authors show that this variant affects the gene expression of the tumour suppressor gene *Cdkn2b*.

## Graphical abstract

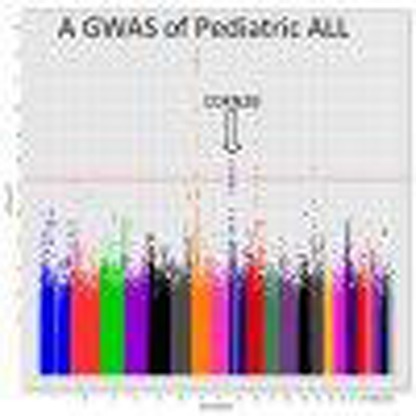

Acute lymphoblastic leukaemia (ALL) is the most common paediatric malignancy, with B-cell precursor ALL (BCP-ALL) accounting for ∼85% of cases^1^. BCP-ALL is characterized by recurring somatic chromosomal abnormalities, many of which are important for diagnosis and risk stratification. Many of the translocations in BCP-ALL, such as the t(12;21)(p13;q22)[ETV6-RUNX1] and *MLL* rearrangements, involve transcriptional regulators of haematopoiesis and are important alterations that precede leukaemogenesis. However, the observation that these abnormalities can be found years before leukaemia onset and are generally not sufficient to induce disease in experimental models^2^,^3^ suggests the involvement of additional genetic or epigenetic susceptibility factors, some of which may be germline.

Overall, it has been estimated that the heritability of paediatric BCP-ALL is 24% (ref. 4). Within the last 10 years a number of loci have been identified by genome-wide association study (GWAS), which have shed considerable light on the genetics of BCP-ALL predisposition. These include variants in *ARID5B*^5^,^6^, *IKZF1* (ref. 6), CEBPE^6^, *BMI1-PIP42KA*^7^, *CDKN2A*^8^, *TP63* (ref. 9) and *GATA3* (refs 10, 11), all of which have odds ratios (ORs) ranging from 1.23 to 1.91. Although their effect sizes are large relative to those of many of the variants identified for other complex diseases, these susceptibility loci explain only 8% of the genetic contribution to childhood BCP-ALL risk^4^. In addition, the mechanisms by which these variants contribute to disease remain to be elucidated.

In this study, to discover additional paediatric BCP-ALL risk variants, we perform a meta-analysis of four paediatric ALL GWAS comprising 1,210 cases and 4,144 controls of European ancestry^9^,^12^,^13^,^14^,^15^. We discover a locus at 9p21.3 tagged by rs77728904, which is independent from the previously reported risk locus in this region tagged by rs3731217 (ref. 8), and we replicate this association in 977 cases and 1,399 controls. To fine-map the associated region, we perform an association study in African Americans (AAs) and find that of single-nucleotide polymorphisms (SNPs) in this region that are both associated with BCP-ALL in Europeans and correlated with rs77728904, only rs662463 is significant in AAs, suggesting that it may be the disease-associated variant tagged by this locus. Functional analysis demonstrates that rs662463 is a *cis*-expression quantitative trait locus (*cis*-eQTL) for *CDKN2B* and disrupts a transcription factor-binding site (TFBS) for CEBPB, a transcription factor (TF) recurrently mutated in BCP-ALL^16^. In individuals homozygous for the rs662463 protective allele, *CDKN2B* expression is significantly correlated with *CEBPB* expression; however, in individuals carrying one or more risk alleles this correlation is abrogated. Our results suggest that rs662463 is associated with BCP-ALL by modifying the ability of CEBPB to regulate *CDKN2B* expression. We also investigate the function of rs3731217, the previously reported BCP-ALL-associated variant in this region. We find that rs3731217 is associated with alternative splicing of *CDKN2A*, suggesting that rs3731217 is associated with BCP-ALL by influencing the messenger RNA stability of the p16 and p14^ARF^ tumour suppressors encoded by this gene. In summary, we conclude that common inherited genetic variation at 9p21.3 is associated with risk for BCP-ALL by modulating the regulation of *CDKN2B* and *CDKN2A* expression. Our results not only shed light on genetic variation predisposing towards BCP-ALL, but also suggest a path forward moving from GWAS associations to underlying mechanism.

## Results

### Identification of a paediatric BCP-ALL locus at 9p21.3

After performing quality control (QC) measures and imputation of four paediatric BCP-ALL data sets^17^, we interrogated 6,784,687 SNPs with minor allele frequencies (MAFs) >1% in 1,210 cases and 4,144 controls of European ancestry (Supplementary Figs 1–3). Each SNP was assessed for disease association in the discovery data sets using an additive allele-dosage logistic regression model and then meta-analysed. We confirmed previously reported associations between BCP-ALL and *IKZF1* at 7p12.2 (refs 5, 6), *ARID5B* at 10q21.2 (refs 5, 6), *CEBPE* at 14q11.2 (ref. 6), *BMI1-PIP4K2A* at 10p12.31-12.2 (refs 7,10,18), *CDKN2A* at 9p21.3 (ref. 8) and *TP63* at 3q28 (ref. 9) (Supplementary Table 1).

**Figure 1:**
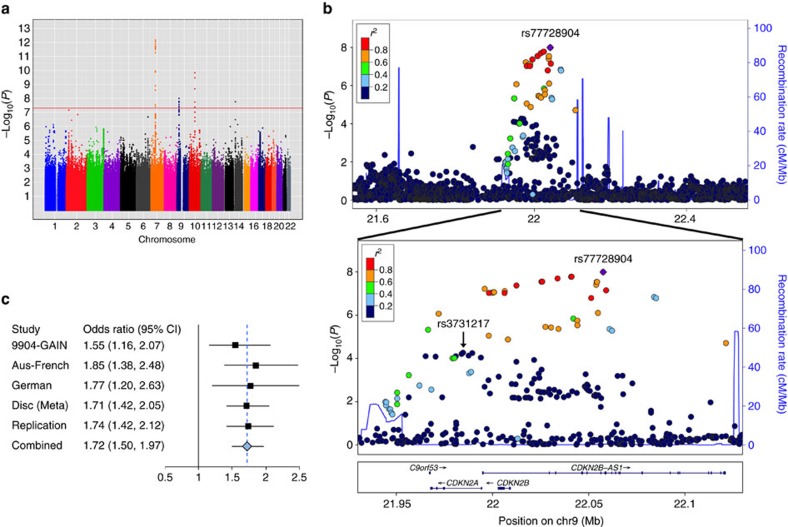
Meta-analysis results for paediatric BCP-ALL. (**a**) Manhattan plot of associations for the discovery GWAS of 1,210 cases and 4,144 controls from four independent studies. The red line denotes the threshold for genome-wide significance. Peaks surpassing this threshold are found at the following: 7p12.2 (*IKZF1*), 10q21.2 (*ARID5B*) and 9p21.3 (*CDKN2*). (**b**) The top panel is the regional LocusZoom plot of the 9p21.3 locus extending 500 kb on either side of the index SNP, rs77728904 (shown in purple), and including all genotyped and imputed SNPs with MAF >0.01. The bottom panel is zoomed in (indicated by the solid black lines between plots) to include only the *CDKN2* locus and surrounding recombination peaks. The *r*^2^ (visualized by colour) demonstrates that rs77728904 and rs3731217 are in independent linkage blocks between the recombination peaks. *r*^2^ for all SNPs in both panels is shown relative to rs77728904. (**c**) Forest plot of OR with 95% confidence intervals (CIs) for rs77728904 in each of the studies comprising the discovery meta-analysis (Disc (Meta)), the full meta-analysis, the replication analysis and the combined analysis. Squares represent the OR; horizontal lines represent the CI; the solid vertical line represents OR=1; the blue dashed vertical line represents the OR in the combined discovery+replication sets.

We discovered a novel BCP-ALL susceptibility locus at 9p21.3 indexed by rs77728904 (*P*_discovery_=1.02 × 10^−8^, OR=1.71, 95% confidence interval=1.42–2.05; Table 1, Fig. 1, Supplementary Tables 2 and 3, and Supplementary Data 1), which we replicated in an independent set of 977 cases and 1,399 controls of European ancestry (*P*_replication_=6.28 × 10^−8^; *P*_combined_=3.32 × 10^−15^, OR=1.72, 95% confidence interval=1.50–1.97). This risk locus (defined as all 1,000 Genomes Phase 1 SNPs in linkage disequilibrium (LD) with rs77728904 (*r*^2^_EUR_>0.6); Table 1 and Supplementary Data 1) spans *CDKN2B* and lies within the first eight introns of *ANRIL* (antisense non-coding RNA in the INK4A locus), a long non-coding RNA that acts as a negative regulator of gene expression^19^ (Fig. 1b).

rs77728904 is not in LD with rs3731217, the *CDKN2A* SNP previously reported as associated with BCP-ALL^8^ (*r*^2^_EUR_=0.015)^17^, and its association with BCP-ALL remains significant after conditioning on rs3731217 (*P*_discovery-conditional_=1.25 × 10^−7^; *P*_combined-conditional_=9.34 × 10^−13^; Table 1). Similarly, rs3731217 retains its significance after conditioning on rs77728904 (Supplementary Table 4). These results demonstrate that the rs77728904-tagged locus is associated with BCP-ALL independently of rs3731217.

The discovery set consisted predominantly of BCP-ALL cases with either an *ETV6-RUNX1* rearrangement (t(12;21)) or double trisomy of chromosomes 4 and 10/high hyperdiploid (>50 chromosomes) tumour karyotype (DT/HHD)^9^,^12^,^13^,^14^. The replication set consisted of a heterogeneous group of BCP-ALL cases, most with neither a t(12;21) translocation nor a DT/HHD tumour karyotype^7^,^14^,^18^. The influence of rs77728904 on BCP-ALL did not differ between BCP-ALL subtypes within the discovery set (Cochran's *P*_heterogeneity_=0.606; Supplementary Table 5) nor did it differ between the discovery and replication studies (Cochran's *P*_heterogeneity_=0.858). Thus, rs77728904 appears to influence paediatric BCP-ALL risk irrespective of cytogenetic subtype.

To refine the risk locus tagged by rs77728904, we leveraged the reduced LD in admixed populations by testing the association between BCP-ALL and all 26 SNPs in the rs77728904-tagged risk locus in AAs and Hispanic Americans (HAs) (203/1,363 AA and 391/1,008 HA cases/controls)^7^. Whereas nearly all SNPs were associated with BCP-ALL in HAs (*P*_nominal_<0.05) and had ORs similar to those in European Americans (EAs), only one variant, rs662463, retained its significance in AAs (*P*_nominal_=0.003; Table 2).

**Figure 2:**
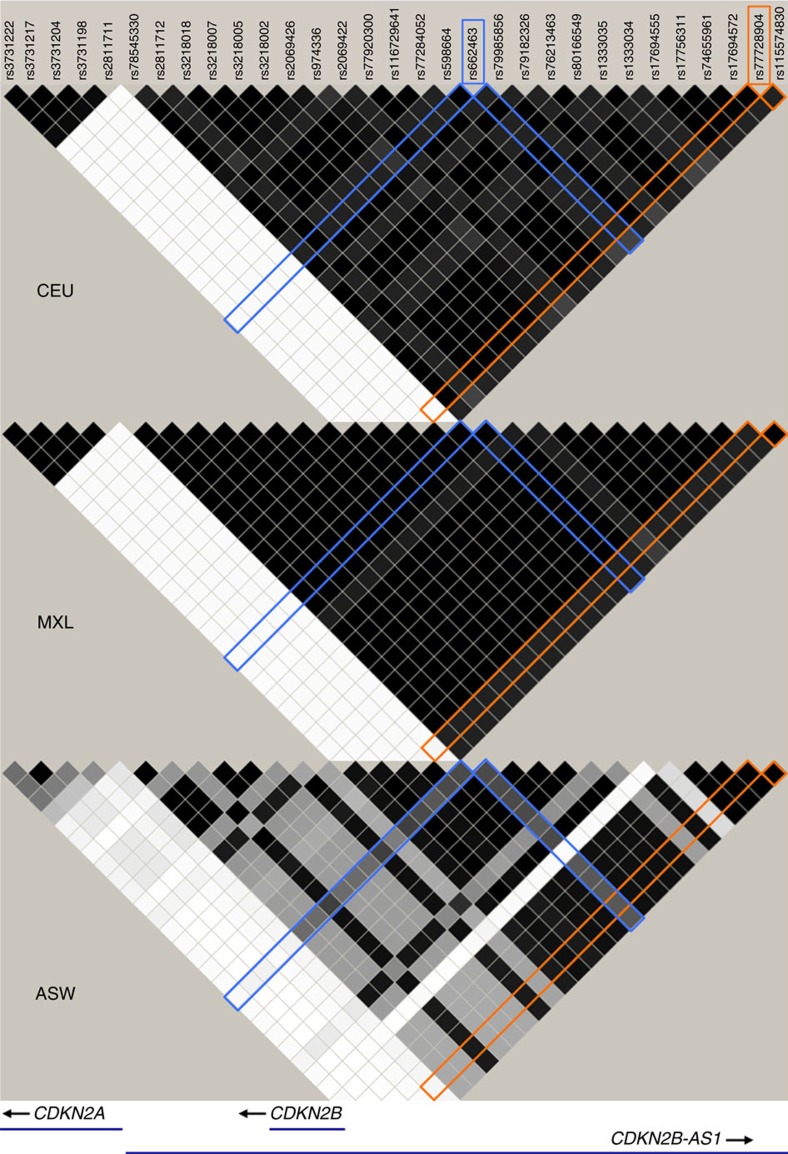
Haploview LD map of the 9p21.3 locus showing differences in the LD structure by ancestry in 1,000 Genomes Phase 1 populations. LD is reported as *r*^2^ with *r*^2^=0.01 white; 0.01<*r*^2^<1 shades of grey; *r*^2^=1 black. ASW, African American ancestry; CEU, European ancestry; MXL, Hispanic ancestry. rs77728904 is highlighted in orange and rs662463 is highlighted in blue.

Although we did not detect an association in AAs between BCP-ALL and either rs77728904 or any other SNP in the rs77728904-tagged locus except for rs662463, we were powered to do so; assuming the effect size of each SNP in AAs was the same as in EAs (Power_one-sided_=0.98 for rs77728904; Supplementary Table 6). In addition, we observed that there is remarkably lower LD in this region in AAs (based on 1000 Genomes ASW population) as compared with that in EAs (*r*^2^_rs662463/rs77728904-EUR_=0.70 versus *r*^2^_rs662463/rs77728904-ASW_=0.58; Fig. 2 and Supplementary Table 6). To determine whether the LD structure in AAs is consistent with our finding in AAs that rs662463 is associated with BCP-ALL but rs77728904 is not, we generated 1,000 simulated data sets using haplotypes from the 1,000 Genomes ASW panel that exactly matched both (1) the number of cases and controls, and (2) the rs662463 genotype frequencies of the original data set. We found that the association *P*-value for rs77728904 was less significant in 26% of the simulated data sets than the association *P*-value for rs77728904 in the original data set. This indicates that it is common to find in AAs that the association between BCP-ALL and rs77728904 is not significant even when the association between BCP-ALL and rs662463 is significant.

Taken together, these results suggest that rs662463 is the causal variant tagged by rs77728904.

### Functional analysis of the rs77728904-tagged risk locus

**Figure 3:**
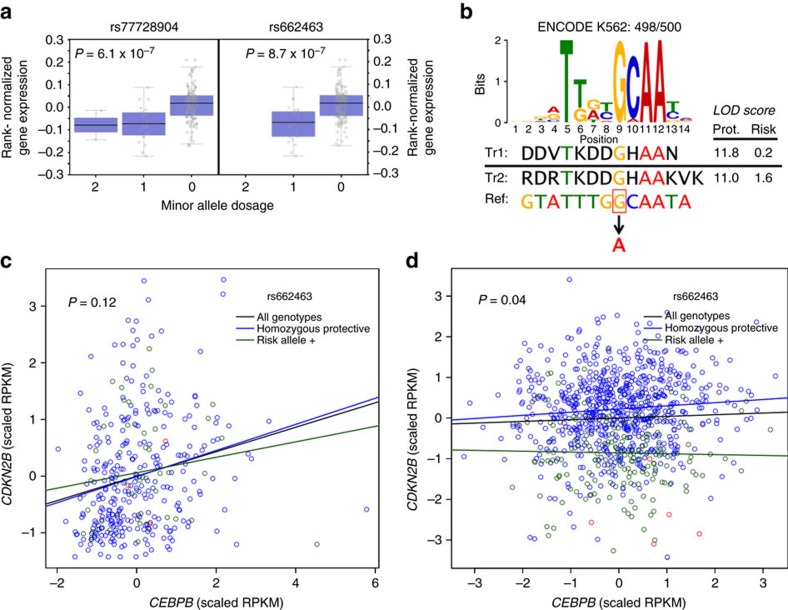
Multi-ethnic and functional analysis suggesting rs662463 is the causative BCP-ALL SNP tagged by the rs77728904-defined locus. (**a**) eQTL analysis from GTEx^22^ in whole blood showing the association of *CDKN2B* expression with rs77728904 and rs662463 genotypes. For both SNPs, the minor allele is the risk allele. Each grey circle represents an individual. Each box plot shows the median rank normalized gene expression (black horizontal line), the first through third quartiles (purple box) and 1.5 × the interquartile range (whiskers). (**b**) Motifs derived from ChIP data showing the effect of rs662463 on CEBPB binding. The genomic sequence (Ref) surrounding rs662463 is shown below the CEBPB-binding site motif logo for K562 cells from Factorbook^27^, with the reference protective G-allele boxed and the risk A-allele in red. The CEBPB motif logo represents the position weight matrix (PWM) for each base. The PWM LOD scores calculated by HaploReg^24^ from TRANSFAC^28^ for two CEBPB-binding motifs (‘Tr1' or TRANSFAC accession M00912 and ‘Tr2' or TRANSFAC accession M00109) including either the protective or the risk allele of rs662463 demonstrate that the risk allele disrupts CEBPB binding. (**c**) RNA-seq analysis in European ancestry LCLs^29^, suggesting that the rs662463 genotype influences the correlation between *CDKN2B* and *CEBPB* expression, and that the rs662463 risk allele is associated with lower *CDKN2B* expression. Shown are the best-fit lines overall (black line), for LCLs homozygous for the protective allele (blue line) and for LCLs with at least one copy (one copy: green circles and two copies: red circles) of the risk allele (green line). (**d**) RNA-seq analysis in whole blood from an independent set of European ancestry individuals^23^, demonstrating that the rs662463 genotype significantly influences the correlation between *CDKN2B* and *CEBPB* expression. The rs662463 risk allele attenuates this correlation and is associated with lower *CDKN2B* expression. Shown are the best-fit lines overall (black line), for individuals homozygous for the protective allele (blue line) and for individuals with at least one copy (one copy: green circles and two copies: red circles) of the risk allele (green line).

As the functional consequence of variants identified by GWAS is often the regulation of gene expression^20^ and tissue context is a major determinant of gene expression^21^, we employed genotype and RNA sequencing (RNA-seq) data in whole blood from the GTEx (Genotype-Tissue Expression) project^22^, to investigate the association between the rs77728904-tagged risk locus and gene expression. We found that all SNPs in the locus found in GTEx are *cis*-eQTLs for *CDKN2B*, with increasing dosage of the risk allele associated with decreasing levels of *CDKN2B* mRNA (*P*_additive,rs77728904_=6.1 × 10^−7^ and *P*_additive,rs662463_=8.7 × 10^−7^; Fig. 3a and Supplementary Table 7). We confirmed that both rs77728904 and rs662463 are *cis*-eQTLs for *CDKN2B* in whole blood using an independent data set of genotype and RNA-seq data from 922 individuals of European descent (*P*_additive,rs77728904_<2 × 10^−16^ and *P*_additive,rs662463_<2 × 10^−16^)^23^.

Data from HaploReg^24^ and RegulomeDB^25^ show that the entire 9p21.3 locus is rife with regulatory motifs; a complete list of functional annotation has been provided in Supplementary Data 2–4. Of SNPs in the locus, rs662463 is not only a *cis*-eQTL for *CDKN2B* but it is also the most functionally compelling variant based on ENCODE annotation^26^: by DNase sequencing, it is in a DNase hypersensitivity peak in 72 cell lines; by chromatin immunoprecipitation sequencing (ChIP-seq), it is bound by 14 TFs; and by position-weight matrix score, the presence of the risk allele is predicted to disrupt four of the nine TFBSs in which rs662463 resides (CEBPB, HNF1, SOX and P300)^26^.

To explore the role of rs662463 in the proper tissue context, we evaluated ENCODE ChIP-seq data from lymphoblastoid cell lines (LCLs). We found that rs662463 lies within chromatin marked by H3k9me3, H3k27me3, H4k20me1, H3k4me1, H3k4me3 and H2az modifications, suggesting it resides in a TFBS motif regulating *CDKN2B* expression in BCPs. Intriguingly, the protective G-allele of rs662463 (associated with higher *CDKN2B* expression) is part of a ChIP-seq-validated TFBS for CEBPB (CCAAT/enhancer-binding protein β), a TF important for haematopoietic differentiation that is mutated in some BCP-ALL patients^16^, and substitution by the risk A-allele (associated with lower *CDKN2B* expression) disrupts this binding motif (Fig. 3b)^26^,^27^,^28^.

This confluence of genetic and functional evidence led us to hypothesize that rs662463 regulates *CDKN2B* expression by modifying CEBPB signalling. If so, we surmised that *CDKN2B* and *CEBPB* expression would be correlated in an rs662463-genotype-dependent manner, with the correlation decreasing with increasing risk allele dosage. To investigate the modifying effect of rs662463 genotype on the coexpression of *CEBPB* and *CDKN2B*, we performed a likelihood ratio test (LRT) using genotype and RNA-seq data from 358 European-ancestry LCLs^29^. We observed a positive correlation between the expression of these genes (Spearman's correlation *r*=0.22, *P*_Spearman_=4.3 × 10^−9^). Although we did not find statistically significant evidence for genotype-dependent coexpression (*P*_LRT,one-sided_=0.12), the correlation between *CDKN2B* and *CEBPB* expression was consistent with our hypothesis, exhibiting greater correlation in the homozygous protective genotype group than in the risk allele-containing group (*r*_hom prot_=0.24 and *r*_risk allele+_=0.15; Fig. 3c, Supplementary Fig. 4 and Supplementary Data 5). We then investigated rs662463-genotype-dependent coexpression between *CDKN2B* and *CEBPB* in whole blood using the same set of 922 European-ancestry individuals employed to confirm that rs662463 is a *cis*-eQTL for *CDKN2B*^23^. In this data set, we found that *CDKN2B* and *CEBPB* expression was significantly correlated in individuals homozygous for the rs662463 protective allele (*r*=0.08, *P*_Spearman_=0.02), but not in individuals with one or more risk alleles (*r*=−0.06, *P*_Spearman_=0.41). Moreover, the difference in correlation between these groups was significant (*P*_LRT,one-sided_=0.04; Fig. 3d). Thus, these results from two independent European ancestry data sets provide evidence, suggesting that *CDKN2B* and *CEBPB* expression is correlated in an rs662463-dependent manner, with the rs662463 risk allele associated with lower *CDKN2B* expression. In 326 African-ancestry LCLs, however, we found neither a significant correlation between *CDKN2B* and *CEBPB* expression (*P*_Spearman_=0.42) nor a significant influence of rs662463 genotype on their coexpression (*P*_LRT,two-sided_=0.63; Supplementary Data 6)^30^.

In European-ancestry LCLs, *CDKN2B* expression was also correlated with the expression of other TFs with TFBSs containing SNPs in the 9p21.3 locus, many with roles in lymphopoiesis. We investigated the coexpression between *CDKN2B* and all of these TFs (Supplementary Data 5). We found that only *JUND*, a component of the AP-1 TF, whose canonical TFBS is only two bases away from that of *CEBPB*, was nominally significantly coexpressed with *CDKN2B* in a genotype-dependent manner. Surprisingly, the SNP mediating this coexpression was again rs662463 (*P*_LRT,one-sided_=0.004; Supplementary Fig. 4). As *JUND* expression was not measured in Africans, we investigated coexpression between *CDKN2B* and *cJUN*, a *JUND* family member whose expression is highly correlated with that of *JUND* in Europeans (*r*=0.54, *P*_Spearman_<10^−12^). We found that although *cJUN* and *CDKN2B* expression was correlated in Africans in an rs662463-genotype-dependent manner (*P*_LRT,two-sided_=0.02), the risk allele was associated with higher *CDKN2B* expression, which is inconsistent with our results in European ancestry individuals and our eQTL results. In addition, in the European-ancestry whole blood data set we found that although *JUND* and *CDKN2B* expression was significantly correlated (*r*=−0.09, *P*_Spearman_=0.0058), there was no evidence that this correlation was modified by rs662463 genotype (*P*_LRT,two-sided_=0.67). Coexpression results between *CDKN2B* and all TFs with TFBSs containing SNPs in the p21.3 locus in African-ancestry LCLs are presented in Supplementary Data 6.

Taken together, our integrated genetic and functional analysis of multi-ethnic GWAS, eQTL and TF binding data suggests a model whereby rs662463 predisposes to BCP-ALL by regulating *CDKN2B* expression through CEBPB signalling. In individuals of European ancestry homozygous for the rs662463 protective G-allele, CEBPB signalling positively regulates *CDKN2B* expression. In European-ancestry individuals harbouring at least one risk A-allele, however, CEBPB binding is disrupted, thereby attenuating the influence of CEBPB signalling on *CDKN2B* transcriptional regulation and resulting in lower levels of *CDKN2B* expression. These data underscore the complexity of the relationship between this locus and BCP-ALL, and also highlight possible ancestry-based differences in molecular aetiology.

### Functional analysis of rs3731217

**Figure 4:**
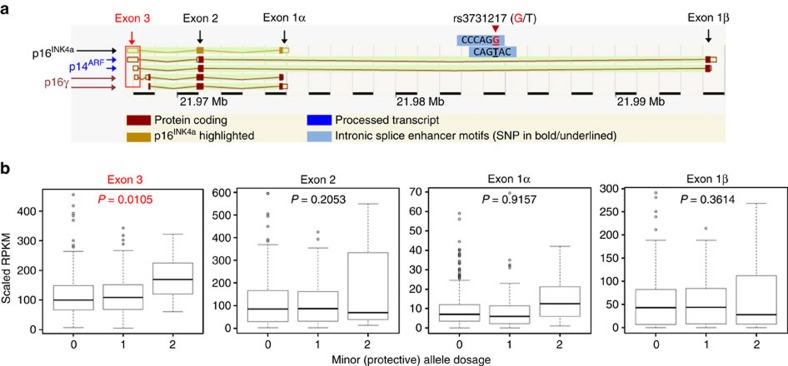
Functional analysis demonstrating rs3731217 is associated with *CDKN2A* exon 3 usage. (**a**) Cartoon showing the major protein isoforms encoded by *CDKN2A*: p14^ARF^ (blue arrows), p16γ (red arrows) and p16^INK4a^ (black arrow). The four main exons are labelled with exon 3 surrounded by a red box. rs3731217 is located in two overlapping intronic splicing elements between exon 1α and exon 1β. The image is modified from AceView^70^. (**b**) RNA-seq data from LCLs correlating exon usage with rs3731217 genotype showing that exon 3 usage is significantly associated with the protective G-allele (0, 1 and 2 on the *x* axis refer to G-allele dosage). Each box plot shows the median rank normalized gene expression (black horizontal line), the first through third quartiles (box) and 1.5 × the interquartile range (whiskers).

We also investigated the functional basis for the association between BCP-ALL and rs3731217, the SNP intronic to *CDKN2A* at 9p21.3 previously found to be associated with BCP-ALL. Consistent with other studies^8^, we found no evidence that rs3731217 is a *cis*-eQTL for *CDKN2A.* However, we observed that the alleles of this SNP create two overlapping *cis*-acting intronic splice enhancer motifs^31^ (CCCAG**G** and CAG**T**AC; Fig. 4a and Supplementary Fig. 5), suggesting rs3731217 may regulate alternative splicing of *CDKN2A*. We assessed the relationship between *CDKN2A* exon usage and rs3731217 using genotype and RNA-seq data from 501 LCLs^29^. We found that the minor G-allele, which is protective against BCP-ALL^8^, is associated with increased expression of exon 3, containing the 3′-untranslated region for this gene (*P*_additive_=0.01; Fig. 4b). *CDKN2A* encodes three proteins that function as tumour suppressors: p16^INK4a^ and p16γ, which regulate cell cycle progression^32^,^33^, and p14^ARF^, which stabilizes p53 by binding MDM2 (ref. 34). As stable translation is dependent on the presence of a 3′-untranslated region^35^, these data may suggest increasing dosage of the rs3731217 G-allele is associated with protection against BCP-ALL by promoting transcript isoforms of *CDKN2A* containing exon 3, resulting in higher protein levels of the tumour suppressors encoded by this gene.

## Discussion

It has long been recognized that *CDKN2* at chromosome 9p21.3 is a common site for somatic mutation acquisition in BCP-ALL^36^,^37^,^38^. It was also shown in recent times that a germline variant in *CDKN2A* (rs3731217) influences risk for BCP-ALL. Here we report an independent risk locus tagged by rs77728904 implicating *CDKN2B*, which encodes the tumour suppressor p15, in the aetiology of BCP-ALL.

Using independent but complementary genetic and bioinformatic/functional strategies, our results suggest that rs662463 is the causal BCP-ALL variant tagged by the locus we discovered. Furthermore, our results imply that the rs662463 risk allele influences *CDKN2B* expression by disrupting a TFBS for CEBPB, although we observed evidence for this mechanism only in individuals of European ancestry. Thus, we speculate that the increased risk for BCP-ALL among individuals with the rs662463 risk allele results from diminished levels of the p15 tumour suppressor as compared with individuals with the protective allele, as a consequence of attenuated CEBPB signalling. Undoubtedly, many factors influence *CDKN2B* expression, making it unlikely that there would be a strong correlation between any one factor and *CDKN2B* expression. Hence, observing a consistent—albeit modest—rs662463 genotype-dependent relationship between *CEBPB* and *CDKN2B* expression in two independent data sets is compelling and deserving of further study.

Although GWAS results identify regions associated with disease, it is often challenging to progress from tagging SNP to causal variant, thereby limiting opportunities to investigate the biological basis for the association. Our results suggest that testing for genotype-dependent coexpression between TFs and eQTL target genes may be an efficient method to prioritize candidate variants, thereby facilitating hypothesis-driven functional follow-up studies.

We also obtained evidence to suggest that the previously reported BCP-ALL risk variant in this region, rs3731217, is associated with BCP-ALL by regulating alternative splicing of *CDKN2A*, which we hypothesize is associated with differences in the translation of the p16 and p14^ARF^ tumour suppressors encoded by this gene. There are several examples of somatic mutations that disrupt the normal regulation of splicing in cancer^39^,^40^. Likewise, rare germline mutations in *CDKN2A* have been found that disrupt normal splicing of this gene and are associated with familial melanoma^41^,^42^.

Thus, our results reveal previously unsuspected complexity between the association of BCP-ALL and the *CDKN2* locus. The remarkable confluence of germline susceptibility variants and somatic mutations point towards the central importance of this region in the aetiology of this disease and warrant further investigation of its role in the regulation of benign and malignant haematopoiesis.

## Methods

### Discovery analysis data sets

The discovery meta-analysis was performed using data from four GWAS consisting of 1,210 paediatric BCP-ALL cases and 4,144 controls of European ancestry (Supplementary Figs 1 and 2)^9^,^12^,^13^,^15^. The GWAS data sets are referred to as the ‘9,904-GAIN' data set, the ‘Aus–French' data set and the ‘German data set', and are described in detail below.

Cases for the 9,904-GAIN data set were 568 children of European ancestry with BCP-ALL treated on The Children's Oncology Group (COG) P9904 protocol ( https://members.childrensoncologygroup.org/Mtg/bookreports/Denver/reports/9904_Fall07SPR_FinalReport.pdf)^14^,^43^. All were diagnosed between the ages of 1 and 9 years (median=3.8 years of age; mean=4.2 years of age; s.d.=1.8 years). Three hundred and twenty cases were males and 248 were females. Two hundred and seventy-five cases (48%) had the t(12;21) *ETV6-RUNX1* translocation tumour karyotype and 293 (52%) had the double DT tumor karyotype. Controls were obtained separately and included 1,014 healthy individuals (464 males and 550 females) from the Genetic Association Information Network (GAIN) Consortium schizophrenia study cohort^44^. Permission to use this data set was obtained from dbGaP (phs000021.v1.p1). Cases and controls were genotyped separately by COG and the Broad Institute, respectively, using the Affymetrix Genome-Wide Human SNP Array 6.0 (Santa Clara, CA) and called using the Birdseed-v2 algorithm^45^. We performed stringent QC before imputation (described below). Twenty individuals (20 cases and 0 controls) failed genotyping (missingness>2%) and 16 individuals (16 cases and 0 controls) were removed for high inbreeding coefficients (|F|>0.05). One hundred and fifty-one population outliers (95 cases and 56 controls) were also removed. Following QC, 437 cases and 958 controls remained and were included in the discovery meta-analysis.

The French data set, described previously, consisted of 223 paediatric BCP-ALL cases and 1,542 controls ascertained through the ESCALE (Etude Sur les Cancers et les Leucémies de l'Enfant) study^12^,^13^. Cases were all diagnosed before age 15 years and were identified through the French National Registry of Childhood Hematopoietic Malignancies. Eighty-one cases (36%) were of the *ETV6-RUNX1* molecular subtype, 123 cases (55%) were of the HHD subtype and 21 (9%) cases were hyperdiploid (47–50 chromosomes). Two cases had both the *ETV6-RUNX1* rearrangement and were HHD. Controls were healthy French adults of European descent from the SU.VI.MAX study^46^. Cases were genotyped using the Illumina Infinium Human CNV370-Quad BeadChip (San Diego, CA) and controls were genotyped using the Illumina Infinium Human-Hap300 (317 K) BeadChip, both by the Centre National du Génotypage^13^.

The Australian data set consisted of 142 paediatric BCP-ALL cases and 1,229 controls obtained through the Aus–ALL Consortium, as described^12^,^15^. Fifty-two cases (37%) were of the *ETV6-RUNX1* molecular subtype, 28 cases (20%) were of the DT subtype and the remaining 62 (43%) cases had >46 chromosomes. All controls and 67 cases were genotyped using the Illumina Infinium Human610-Quad BeadChip; 75 cases were genotyped using the Illumina Infinium Human CNV370-Quad BeadChip.

As the French and Aus–ALL data sets are both part of the Childhood Leukemia International Consortium^47^, we merged them to create the combined ‘Aus–French' data set, from which 11 cases and 59 controls were removed following QC before imputation (described below). The remaining 354 cases (129 *ETV6-RUNX1*, 146 HHD, 21 hyperdiploid and 60 >46 chromosomes) and 2,712 controls were included in the discovery meta-analysis.

The German data set consisted of 491 paediatric BCP-ALL cases, all with the *ETV6-RUNX1* rearrangement, and 483 cancer-free controls, all of European ancestry^9^. Seventy-two cases and 9 controls were removed as previously described^9^. The remaining 419 cases and 474 controls were included in the discovery meta-analysis. Cases were obtained through the Austrian-German-Italian-Swiss multicentre clinical trial AIEOP-BFM ALL 2000 and controls were obtained through the German popgen biobank^9^,^48^. Cases and controls were genotyped together as part of the German National Genome Research Network GWAS initiative by Affymetrix (South San Francisco, CA) using the Affymetrix Genome-Wide Human SNP Array 5.0. Additional QC was performed as described below.

This study was approved by the Institutional Review Board of The University of Chicago.

### Discovery GWAS QC and imputation

Before imputation, we performed the following QC measures in PLINK^49^. Samples were removed based on the following: (a) call rate <0.98, (b) ambiguous gender assignment, (c) cryptic relatedness (pi-hat >0.125) and (d) absolute value of the inbreeding coefficient >0.05. SNPs were excluded based on the following: (a) MAF <0.01 (<0.05 for the German data set), (b) genotype missingness rate >0.05 (>0.03 for the German data set), (c) deviation from Hardy-Weinberg equilibrium (*P*_HWE_<1 × 10^−4^), (d) differential missingness between cases and controls (*P*_missingness_<0.05), (e) ambiguous strand assignment and (f) failure to resolve reference genome assembly ambiguities in LiftOver^50^. Population outliers were identified and removed using principal component analysis implemented through EIGENSTRAT^51^, to ensure that all remaining cases and controls were well matched and of European ancestry. A flowchart detailing these QC measures is provided in Supplementary Fig. 1.

To improve the genome-wide coverage of SNPs for association testing, we performed imputation separately for each discovery data set (cases and controls together) using IMPUTE2 v2.1.2.3 (ref. 52) and the 1,000 Genomes Project^17^ Phase 1 v3 reference panel (all ancestries panel, build 37, released March, 2012). Pre-imputation data sets were updated to build 37 using LiftOver^50^. Sample haplotypes were phased by chromosome before imputation using SHAPEIT^53^. Poorly imputed SNPs, defined by info scores <0.3, were excluded from further analysis. The number of SNPs successfully imputed for each study is shown in Supplementary Fig. 2.

### Discovery association analysis

Following imputation, we analysed the association of SNPs with BCP-ALL in SNPTEST^54^ separately for each of the three discovery data sets with a frequentist additive missing data likelihood score test (SNPTEST-method score), which uses the genotype posterior probabilities generated during imputation to account for uncertainty in the genotypes. To control for residual population stratification in each data set, significant principal components (PCs) inferred by EIGENSTRAT^51^ were included as covariates for association testing (PC1 for 9,904-GAIN, PC1 for Aus–French and PC1–PC2 for German). Following association testing in SNPTEST^54^, SNPs were excluded from analysis based on the following: (a) *P*=not available (NA), which occurs when a model cannot be fit to the data; (b) info score for *β*<0.3; (c) MAF <0.01; and (d) deviation from HWE (*P*_HWE_<1 × 10^−4^).

### Discovery meta-analysis

Only SNPs passing genotyping and imputation QC and shared among all three data sets following post SNPTEST^54^ QC were included in the meta-analysis (Supplementary Fig. 2). The meta-analysis was performed using a fixed-effects model with inverse variance weighting, as implemented in METAL^55^. SNPs were excluded based on high heterogeneity among data sets (*I*^2^>50%)^56^. Genome-wide significance was defined as *P*<5 × 10^−8^. We assessed inflation of the meta-analysis test statistics by examining quantile–quantile plots of the expected and observed distributions of *P*-values and by estimating *λ*_GC_^57^ (Supplementary Fig. 3). We found no evidence for systematic genotyping differences between cases and controls.

Regions associated with BCP-ALL were visualized using LocusZoom^58^ by plotting the −log_10_ of the meta-analysis *P*-values and local recombination rate. LD patterns were plotted with Haploview^59^ using LD estimates from the 1,000 Genomes Phase 1 CEU, ASW and MXL populations^17^ (NCBI build 37 assembly).

### Fine-mapping of the 9p21.3 locus

We mapped the association with BCP-ALL of all SNPs within 500 kb of the index SNP rs77728904. The significance of association dropped precipitously as a function of *r*^2^ relative to rs77728904. Consequently, we chose to replicate and further analyse the SNPs listed in HaploReg^24^ in LD (*r*^2^_EUR_>0.60 in the 1,000 Genomes Phase 1 data set) with rs77728904 (Table 1). Results for all SNPs with *r*^2^>0.20 relative to rs77728904 are reported in Supplementary Data 1.

### Replication set analysis

The association between paediatric BCP-ALL and the novel 9p21.3 locus was validated in an independent replication set of 977 cases and 1,399 controls of European ancestry. Cases comprise two cohorts of paediatric BCP-ALL patients: one consisting of patients enroled on the COG-P9905 protocol^14^ (‘9,905 cohort') genotyped on the Affymetrix 6.0 array (520 cases) and the other consisting of patients enroled on either the St Jude Total Therapy XIIIB/XV (‘SJ') protocol or the COG-P9906 protocol genotyped on the Affymetrix GeneChip Human Mapping 500K array and collectively referred to as the ‘SJ cohort' (457 cases)^7^,^18^. Controls were from the Multi-Ethnic Study of Atherosclerosis (MESA) study (dbGaP phs000209.v9)^60^ genotyped on the Affymetrix 6.0 array. Permission to the use the MESA study was obtained from dbGaP.

Variants not directly genotyped were imputed using IMPUTE2 v2.1.2.3 (ref. 52) as described above. Association was tested in PLINK using a logistic regression model that fit an additive allele-dosage model for the SNP along with the top four eigenvectors from principal component analysis. SNPs with one-sided *P*<0.05 and the same risk allele as in the discovery set were considered validated.

### Multi-ethnic set analysis

To identify the functional SNP tagged by rs77728904, we investigated the patterns of association between paediatric BCP-ALL and the 9p21.3 locus in patients of HA and AA ancestry. AA cases consisted of 203 individuals from the SJ (*n*=114), 9,904 (*n*=38) and 9,905 (*n*=51) data sets, as previously described^7^, and 1,363 AA controls from the MESA data set^60^. HA cases consisted of 391 individuals from the SJ (*n*=86), 9,904 (*n*=123) and 9,905 (*n*=182) data sets, as previously described^7^, and 1,008 HA MESA controls. Ancestry for both cases and controls was determined using STRUCTURE^61^,^62^ (version 2.2.3) with HapMap CEU, YRI, CHB/JPT and indigenous Native Americans^63^ as reference populations. European ancestry was defined as >95% CEU, African ancestry was defined as >70% YRI and Hispanic ancestry as >10% Native Americans>YRI.

Genotyping platforms are specified above and imputation was performed as described. Association testing was performed in PLINK, using an additive allele-dosage logistic regression model that fit genetic ancestries, defined by the first four principle components. SNPs with one-sided *P*<0.05 and the same risk allele as in the discovery set were considered validated.

### Conditional tests of independence

To assess the independence of the association signal for SNPs in the rs77728904-tagged locus from the previously reported *CDKN2A* SNP, rs3731217 (ref. 8), in the discovery cohort, we used SNPTEST^54^ (-condition_on) to test each of the 26 SNPs in the novel locus for association with BCP-ALL conditional on rs3731217. For the replication set (Table 1) and admixed populations (Table 2), we performed logistic regression in PLINK^49^ using rs3731217 as a covariate.

We also tested the association of rs3731217 with BCP-ALL using SNPTEST^54^ (-condition_on) when conditioning on either the index SNP (rs77728904) or rs662463. These analyses were performed separately for each of the three discovery data sets and also for the discovery meta-analysis (Supplementary Table 4).

### Simulation analysis of the 9p21.3 locus in AAs

Of all SNPs significant in the 9p21.3 locus in EAs, only rs662463 was significant in AAs (*P*_rs662463_=0.003 versus *P*_rs77728904_=0.403). To confirm that this difference in significance was consistent with the LD structure of the region in AAs, we performed simulations (Supplementary Table 6). To create each simulated data set, we sampled without replacement rs77728904-rs662463 haplotypes from the 122 individuals in the 1,000 Genomes ASW sample until 203 case and 1,363 control haplotype pairs were collected. The sampling was performed, to generate the same rs662463 genotype counts as in the original 203 AA cases and 1,363 AA controls. We then tested the association between rs77728904 and BCP-ALL in the simulated data set using a *χ*^2^-test of homogeneity. We repeated the sampling and association testing 1,000 times and recorded the *P*-values. We then estimated the probability of observing an association *P*-value at rs77728904 with the same or greater significance as observed in our AA sample.

### *Cis*-eQTL analysis of the 9p21.3 locus

To determine whether SNPs in the 9p21.3 locus were *cis*-eQTLs for *CDKN2B* or other local genes, we queried the GTEx project database^22^ (Supplementary Table 7). Within GTEx, we accounted for tissue context by investigating eQTLs in whole blood^22^. To confirm our findings, we replicated our observation that rs77728904 and rs662463 were *cis*-eQTLs for *CDKN2B* in an independent data set of eQTLs in whole blood from 922 individuals^23^. All individuals used in the *cis*-eQTL analysis were of European ancestry.

### Bioinformatics analysis of the 9p21.3 locus

To explore the function of SNPs in the 9p21.3 locus, we used the web-based Haploreg^24^ and RegulomeDB^25^,^64^ resources that integrate SNP frequency and LD information from 1,000 Genomes Phase 1 individuals and functional annotation from a variety of biological databases, including motif instances and enhancer annotations from the ENCODE^26^ and Roadmap Epigenetics^65^ Projects.

### *CDKN2B* and *CEBPB* coexpression analysis

Our genetic results suggested that rs662463 may be the causal variant tagged by the 9p21.3 locus. As rs662463 is a *cis*-eQTL for *CDKN2B* and is a critical residue in a number of TFBSs (Supplementary Data 2–4), we hypothesized that it was associated with BCP-ALL by regulating *CDKN2B* expression in response to TF signalling. To test this, we first used the Haploreg^24^ and RegulomeDB^25^,^64^ databases to determine the set of TFs with TFBSs containing rs662463, whose expression was correlated with *CDKN2B* expression, and we then assessed the dependence of this coexpression on rs662463 genotype.

Of the nine TFs with binding motifs containing rs662463, the binding affinity of four (CEBPB, HNF1, SOX and P300) was substantially altered by rs662463 genotype status based on position-weight matrix analysis and ENCODE ChIP-seq data^26^ (Supplementary Data 2). We employed RNA-seq data in LCLs derived from 358 individuals of European ancestry (1,000 Genomes Phase 1 populations CEU, TSI, FIN and GBR)^29^ and Illumina expression array data derived from LCLs for 326 individuals of African ancestry (HapMap populations YRI, MKK and LWK)^30^, to determine whether the expression of any of these TFs was correlated with *CDKN2B* expression in Europeans and in Africans. We assessed the significance of the correlation using a *t*-test based on Pearson's correlation coefficient. We found that only *CEBPB* expression was correlated with *CDKN2B* expression, but only in Europeans (Spearman's correlation *r*=0.22, *P*_Spearman_=4.3 × 10^−9^; Supplementary Data 5 and 6) and not in Africans.

To determine whether rs662463 genotype status altered the relationship between *CDKN2B* and *CEBPB* expression, we used the European and African expression data sets described above, as well as genotype data from the 1,000 Genomes Project^17^ and HapMap 3 (ref. 66). We assessed separately in Europeans and Africans whether the correlation between the *CDKN2B* and *CEBPB* expression differed by rs662463 genotype class (0 risk alleles versus ≥1 risk allele) by fitting two competing linear regression models: the first model regressed *CDKN2B* expression on *CEBPB* expression and rs662463 genotype class (the reduced model), and the second model regressed *CDKN2B* expression on *CEBPB* expression, rs662463 genotype class and the interaction between *CEBPB* expression and rs662463 genotype class (the full model). The interaction term in the full model allows different correlations between *CDKN2B* and *CEBPB* in each rs662463 genotype class, whereas the reduced model requires the correlation to be the same in both genotype classes. Expression levels were scaled to have mean 0 and variance 1, to allow regression parameters to be interpretable as a difference in correlation. An LRT was used to determine whether the full model fit the data significantly better than the reduced model.

As the homozygous risk genotype class for rs662463 (and all other SNPs in the 9p21.3 locus) is rare, the full model includes only a single interaction term that estimates the difference in the *CDKN2B*–*CEBPB* correlation between the homozygous protective and the risk allele-containing genotype classes. We used these estimates, their s.e. and the inverse variance weighting method, to calculate the population-averaged difference in correlation coefficients between the two genotype classes and to perform population-specific meta-analyses^55^. The results of this analysis are summarized in Supplementary Data 5 and 6.

To reproduce our findings, we repeated this analysis in a second independent data set. We used the same data set of 922 individuals of European ancestry previously employed to confirm that SNPs in the 9p21.3 locus were *cis*-eQTLs for *CDKN2B*. Here, rs662463 was directly genotyped, and *CDKN2B* and *CEBPB* expression was measured by RNA-seq in whole blood^23^. Tests for genotype-dependent coexpression were performed as described.

### Coexpression between *CDKN2B* and other TFs in the 9p21.3 locus

Using the EA and AA LCL data sets described above^29^,^30^, we also investigated genotype-dependent coexpression between *CDKN2B* and every TF that either: (1) possessed a binding motif (defined by JASPER^67^ or TRANSFAC^28^) containing an SNP in the rs77728904-tagged locus or (2) was confirmed by ChIP-seq to bind to a sequence containing an SNP in the locus (Supplementary Data 3 and 4). For the European RNA-seq data, we also required the RPKM (reads per kilobase of transcript per million mapped reads) value^68^ to be >0.01 in >75% of samples for each TF investigated.

We calculated the Spearman's correlation coefficient between *CDKN2B* expression and the expression of each TF, and assessed the significance of these correlation coefficients using both an asymptotic *t* approximation and bootstrapping. For each TF-SNP pair, we used an LRT comparing the full and reduced models, as described above, to determine whether the correlation between *CDKN2B* expression and TF expression varied by SNP genotype in either EAs or AAs.

### Functional analysis of rs3731217

We examined the functional significance of rs3731217 using RNA-seq data for LCLs from the GEUVADIS Consortium^29^ by testing for an association between rs3731217 genotype and exon usage using an additive model. The analysis was conducted in 501 LCLs of European ancestry from the 1,000 Genomes CEU, FIN, GBR and TSI populations. Linear regression was performed on log_2_-transformed RNA expression data and the significance of the association between the SNP genotype and expression was assessed using a *t*-test.

### Statistical analysis

Except when specified, all analyses were performed using R statistical software (version 2.15.1)^69^.

## Additional information

**How to cite this article:** Hungate, E. A. *et al*. A variant at 9p21.3 functionally implicates *CDKN2B* in paediatric B-cell precursor acute lymphoblastic leukaemia aetiology. *Nat. Commun.* 7:10635 doi: 10.1038/ncomms10635 (2016).

## Supplementary Material

Supplementary Information Supplementary Figures 1-5, Supplementary Tables 1-7 and Supplementary References.

Supplementary Data 1 Fine mapping of the 9p21.3 locus using 1000 Genomes27 data. SNPs interrogated were located 500kb upstream and downstream of rs77728904. SNPs reported here lie within this region and are in r2EUR>0.2 with rs77728904.

Supplementary Data 2 Transcription factor binding site motif alterations within the novel 9p21.3 locus based on HaploReg v2 annotation29.

Supplementary Data 3 Functional annotation of SNPs within the novel 9p21.3 locus using HaploReg v229..

Supplementary Data 4 Functional annotation in LCLs of SNPs within the novel 9p21.3 locus based on ENCODE Project30 data using RegulomeDB version 1.137.

Supplementary Data 5 Analysis of co-expression between CDKN2B and TFs with evidence for binding to SNPs within the 9p21.3 locus in LCLs from Europeans17. All TFs with evidence for binding to SNPs in the locus are shown, based on HaploReg v229 and RegulomeDB37 annotation. Correlation difference P values assess the significance of genotype-dependent co-expression.

Supplementary Data 6 Analysis of co-expression between CDKN2B and TFs with evidence for binding to SNPs in the 9p21.3 locus in LCLs from Africans38. All TFs with evidence for binding to SNPs in the locus are shown, based on HaploReg29 and RegulomeDB37 annotation. Correlation difference P values assess the significance of genotype-dependent co-expression.

## Figures and Tables

**Table 1:** Association of SNPs in the 9p21.3 locus* with BCP-ALL in individuals of European ancestry.

**SNP rsID**†	**Position (bp)**‡	**LD (*****r***^**2**^)§	**Alleles**‖	**Discovery meta-analysis (1,210 cases and 4,144 controls)**					**Replication (977 cases and 1,399 controls)**			**Combined**		
				OR (95% CI)*	***P*****-value**		***P***_**het**_¶	***I***^**2**^ **(%)**#	OR (95% CI)*	***P*****-value**		OR (95% CI)*	***P*****-value**	
					Unconditional**	**Conditional**††				Unconditional**	**Conditional**††		Unconditional**	**Conditional**††
rs78545330	21,995,941	0.77	A/T	1.58 (1.34–1.86)	6.04 × 10^−8^	1.06 × 10^−6^	0.480	0	1.57 (1.31–1.89)	1.19 × 10^−6^	1.84 × 10^−5^	1.58 (1.39–1.78)	3.44 × 10^−13^	8.37 × 10^−11^
rs2811712	21,998,035	0.69	G/A	1.43 (1.22–1.68)	8.91 × 10^−6^	1.09 × 10^−4^	0.202	37.5	1.52 (1.34–1.69)	3.89 × 10^−6^	7.60 × 10^−5^	1.47 (1.31–1.65)	1.66 × 10^−10^	3.40 × 10^−8^
rs3218018	21,998,139	0.79	G/T	1.59 (1.34–1.88)	9.39 × 10^−8^	1.42 × 0^−6^	0.548	0	1.58 (1.31–1.91)	1.74 × 10^−6^	4.76 × 10^−5^	1.59 (1.40–1.80)	7.68 × 10^−13^	2.85 × 10^−10^
rs3218007	21,999,800	0.77	T/C	1.57 (1.33–1.84)	9.04 × 10^−8^	1.49 × 10^−6^	0.498	0	1.57 (1.31–1.88)	1.37 × 10^−6^	2.23 × 10^−5^	1.57 (1.39–1.77)	5.86 × 10^−13^	1.42 × 10^−10^
rs3218005	22,000,247	0.77	C/T	1.57 (1.33–1.84)	9.05 × 10^−8^	1.50 × 10^−6^	0.498	0	1.57 (1.31–1.88)	1.37 × 10^−6^	2.23 × 10^−5^	1.57 (1.39–1.77)	5.87 × 10^−13^	1.42 × 10^−10^
rs3218002	22,000,841	0.77	A/G	1.57 (1.33–1.85)	8.65 × 10^−8^	1.45 × 10^−6^	0.491	0	1.57 (1.31–1.88)	1.37 × 10^−6^	2.23 × 10^−5^	1.57 (1.39–1.77)	5.62 × 10^−13^	1.38 × 10^−10^
rs2069426	22,006,273	0.79	T/G	1.59 (1.34–1.88)	9.47 × 10^−8^	1.42 × 10^−6^	0.545	0	1.58 (1.31–1.90)	2.23 × 10^−6^	5.85 × 10^−5^	1.58 (1.39–1.80)	9.93 × 10^−13^	3.52 × 10^−10^
rs974336	22,006,348	0.77	T/C	1.57 (1.33–1.85)	7.67 × 10^−8^	1.25 × 10^−6^	0.514	0	1.60 (1.33–1.92)	4.18 × 10^−7^	7.52 × 10^−6^	1.58 (1.40–1.79)	1.58 × 10^−13^	4.17 × 10^−11^
rs2069422	22,008,026	0.70	G/T	1.44 (1.22–1.69)	1.33 × 10^−5^	1.45 × 10^−4^	0.240	30.0	1.52 (1.33–1.70)	6.53 × 10^−6^	1.91 × 10^−4^	1.47 (1.30–1.66)	4.02 × 10^−10^	1.05 × 10^−7^
rs77920300	22,012,441	0.79	T/C	1.60 (1.35–1.89)	4.26 × 10^−8^	6.50 × 10^−7^	0.583	0	1.57 (1.30–1.89)	2.43 × 10^−6^	6.26 × 10^−5^	1.59 (1.40–1.80)	4.95 × 10^−13^	1.78 × 10^−10^
rs116729641	22,025,432	0.79	A/G	1.61 (1.36–1.90)	2.84 × 10^−8^	4.49 × 10^−7^	0.614	0	1.57 (1.30–1.89)	2.31 × 10^−6^	5.83 × 10^−5^	1.59 (1.40–1.80)	3.16 × 10^−13^	1.17 × 10^−10^
rs77284052	22,025,885	0.79	T/C	1.61 (1.36–1.90)	2.82 × 10^−8^	4.46 × 10^−7^	0.618	0	1.57 (1.30–1.89)	2.31 × 10^−6^	5.83 × 10^−5^	1.59 (1.40–1.80)	3.14 × 10^−13^	1.16 × 10^−10^
rs598664	22,027,551	0.70	C/T	1.46 (1.25–1.72)	3.46 × 10^−6^	4.19 × 10^−5^	0.287	19.9	1.51 (1.26–1.81)	7.91 × 10^−6^	2.21 × 10^−4^	1.48 (1.32–1.67)	1.21 × 10^−10^	3.47 × 10^−8^
rs662463	22,030,438	0.70	A/G	1.46 (1.24–1.71)	3.71 × 10^−6^	4.43 × 10^−5^	0.288	19.7	1.50 (1.25–1.79)	1.16 × 10^−5^	3.03 × 10^−4^	1.48 (1.31–1.66)	1.87 × 10^−10^	4.97 × 10^−8^
rs79985856	22,033,824	0.79	T/C	1.61 (1.37–1.91)	2.03 × 10^−8^	3.29 × 10^−7^	0.657	0	1.56 (1.30–1.89)	2.86 × 10^−6^	7.22 × 10^−5^	1.59 (1.41–1.80)	2.84 × 10^−13^	1.08 × 10^−10^
rs79182326	22,034,267	0.69	T/C	1.45 (1.24–1.70)	4.30 × 10^−6^	5.47 × 10^−5^	0.303	16.3	1.45 (1.21–1.73)	4.72 × 10^−5^	1.00 × 10^−3^	1.45 (1.29–1.63)	8.33 × 10^−10^	1.96 × 10^−7^
rs76213463	22,040,839	0.79	A/G	1.62 (1.37–1.91)	1.68 × 10^−8^	2.76 × 10^−7^	0.636	0	1.56 (1.29–1.88)	3.70 × 10^−6^	8.62 × 10^−5^	1.59 (1.40–1.80)	3.08 × 10^−13^	1.11 × 10^−10^
rs80166549	22,041,155	0.79	G/A	1.62 (1.37–1.91)	1.67 × 10^−8^	2.74 × 10^−7^	0.635	0	1.56 (1.29–1.88)	3.70 × 10^−6^	8.62 × 10^−5^	1.59 (1.40–1.80)	3.06 × 10^−13^	1.10 × 10^−10^
rs1333035	22,044,059	0.69	G/A	1.46 (1.25–1.71)	1.78 × 10^−6^	2.50 × 10^−5^	0.238	30.2	1.50 (1.26–1.78)	6.87 × 10^−6^	1.42 × 10^−4^	1.48 (1.31–1.66)	5.45 × 10^−11^	1.36 × 10^−8^
rs1333034	22,044,122	0.70	C/T	1.47 (1.25–1.72)	2.93 × 10^−6^	3.58 × 10^−5^	0.307	15.3	1.50 (1.26–1.80)	9.04 × 10^−6^	2.34 × 10^−4^	1.48 (1.32–1.67)	1.17 × 10^−10^	3.14 × 10^−8^
rs17694555	22,051,295	0.78	G/A	1.61 (1.35–1.92)	1.61 × 10^−7^	2.24 × 10^−6^	0.458	0	1.59 (1.40–1.78)	1.85 × 10^−6^	4.28 × 10^−5^	1.60 (1.41–1.82)	1.40 × 10^−12^	4.08 × 10^−10^
rs17756311	22,053,895	0.77	A/G	1.63 (1.37–1.94)	3.95 × 10^−8^	7.00 × 10^−7^	0.531	0	1.57 (1.30–1.90)	2.92 × 10^−6^	5.74 × 10^−5^	1.60 (1.41–1.82)	5.66 × 10^−13^	1.81 × 10^−10^
rs74655961	22,054,164	0.77	G/A	1.64 (1.38–1.95)	2.90 × 10^−8^	5.16 × 10^−7^	0.557	0	1.55 (1.28–1.87)	5.85 × 10^−6^	1.11 × 10^−4^	1.60 (1.40–1.82)	8.63 × 10^−13^	2.71 × 10^−10^
rs17694572	22,054,356	0.77	A/G	1.63 (1.37–1.94)	2.89 × 10^−8^	4.98 × 10^−7^	0.526	0	1.54 (1.28–1.87)	6.87 × 10^−6^	1.28 × 10^−4^	1.59 (1.40–1.81)	1.01 × 10^−12^	3.02 × 10^−10^
rs77728904	22,057,530	—	C/A	1.71 (1.42–2.05)	1.02 × 10^−8^	1.25 × 10^−7^	0.687	0	1.74 (1.42–2.12)	6.28 × 10^−8^	1.59 × 10^−6^	1.72 (1.50–1.97)	3.32 × 10^−15^	9.34 × 10^−13^
rs115574830	22,059,061	0.87	A/T	1.61 (1.35–1.92)	7.09 × 10^−8^	8.60 × 10^−7^	0.574	0	1.55 (1.28–1.88)	8.34 × 10^−6^	1.38 × 10^−4^	1.58 (1.39–1.80)	2.83 × 10^−12^	5.32 × 10^−10^

AA, African American; BCP-ALL, B-cell precursor acute lymphoblastic leukaemia; CI, confidence interval; GWAS, genome-wide association study; LD, linkage disequilibrium; OR, odds ratio; SNP, single-nucleotide polymorphism.

Highlighted are SNPs with the smallest *P*-values for each part of the analysis (rs77728904 in blue for the discovery GWAS, the replication analysis and the combined analysis; rs662463 in green for AA in the multi-ethnic analysis).

^*^OR with 95% CI.

^†^SNPs in LD (*r*^2^_EUR_>0.60 in 1,000 Genomes Phase 1 (ref. 17) from HaploReg^24^) with rs77728904 (rs115939893 and rs72654280 were not included, because they were not present in the replication and multi-ethnic analyses; all SNPs in *r*^2^_EUR_>0.2 are listed in Supplementary Table 4).

^‡^Physical positions on chromosome 9 are from NCBI build 37/hg19.

^§^LD (*r*^2^) with the index SNP (rs77728904).

^‖^Minor/major allele.

^¶^Cochran's test of heterogeneity in the studies comprising the discovery meta-analysis.

^#^Percentage of variation in the meta-analysis due to heterogeneity.

^**^Unconditional *P*-values for listed SNPs.

^††^*P* values for listed SNPs when conditioned on rs3731217.

**Table 2:** Association of SNPs in the 9p21.3 locus* with BCP-ALL in HAs and AAs.

**SNP (rsID)**	**Position (bp)**†	**LD (*****r***^**2**^) by population*			**HA (NA>10% and NA>YRI**)‡ **(391 cases and 1,008 controls)**			**AA (YRI>70%)**§ **(203 cases and 1,363 controls)**		
		**EUR**‖	**MXL**¶	**ASW**#	OR (95% CI)**	***P***_**HA**_ **value**		OR (95% CI)**	***P***_**AA**_ **value**	
						**Unconditional**††	**Conditional**‡‡		**Unconditional**††	**Conditional**‡‡
rs78545330	21,995,941	0.77	0.83	0.28	1.51 (1.09–2.09)	0.014	0.014	1.18 (0.92–1.50)	0.185	0.255
rs2811712	21,998,035	0.69	0.83	0.28	1.48 (1.16–1.80)	0.017	0.018	1.15 (0.90–1.40)	0.276	0.329
rs3218018	21,998,139	0.79	0.83	0.86	1.54 (1.08–2.20)	0.017	0.030	1.13 (0.79–1.60)	0.512	0.404
rs3218007	21,999,800	0.77	0.83	0.28	1.47 (1.06–2.03)	0.021	0.023	1.14 (0.89–1.46)	0.288	0.347
rs3218005	22,000,247	0.77	0.83	0.28	1.51 (1.09–2.09)	0.014	0.014	1.14 (0.89–1.47)	0.285	0.342
rs3218002	22,000,841	0.77	0.83	0.28	1.49 (1.08–2.06)	0.016	0.014	1.15 (0.90–1.48)	0.253	0.315
rs2069426	22,006,273	0.79	0.83	0.86	1.51 (1.06–2.16)	0.024	0.035	1.16 (0.82–1.64)	0.409	0.333
rs974336	22,006,348	0.77	0.83	0.30	1.48 (1.07–2.05)	0.017	0.014	1.11 (0.87–1.42)	0.391	0.477
rs2069422	22,008,026	0.70	0.83	0.86	1.47 (1.12–1.82)	0.028	0.042	1.28 (0.92–1.64)	0.179	0.157
rs77920300	22,012,441	0.79	0.83	0.93	1.50 (1.05–2.14)	0.025	0.036	1.12 (0.79–1.58)	0.529	0.401
rs116729641	22,025,432	0.79	0.83	0.86	1.48 (1.04–2.12)	0.029	0.043	1.12 (0.79–1.58)	0.537	0.409
rs77284052	22,025,885	0.79	0.83	0.93	1.48 (1.04–2.12)	0.029	0.043	1.12 (0.79–1.58)	0.533	0.406
rs598664	22,027,551	0.70	0.83	0.93	1.44 (1.02–2.05)	0.038	0.057	1.10 (0.78–1.56)	0.580	0.437
rs662463	22,030,438	0.70	0.83	0.58	1.45 (1.03–2.06)	0.034	0.051	**1.55** (**1.16–2.06)**	**0.003**	**0.002**
rs79985856	22,033,824	0.79	0.83	0.93	1.50 (1.05–2.14)	0.026	0.039	1.14 (0.81–1.62)	0.450	0.332
rs79182326	22,034,267	0.69	0.70	0.93	1.41 (1.00–2.00)	0.049	0.073	1.15 (0.81–1.63)	0.425	0.297
rs76213463	22,040,839	0.79	0.83	0.93	1.50 (1.05–2.15)	0.025	0.036	1.17 (0.83–1.63)	0.364	0.292
rs80166549	22,041,155	0.79	0.83	0.93	1.51 (1.05–2.15)	0.024	0.035	1.14 (0.81–1.59)	0.454	0.379
rs1333035	22,044,059	0.69	0.83	0.32	1.41 (1.02–1.94)	0.037	0.032	1.10 (0.87–1.41)	0.427	0.505
rs1333034	22,044,122	0.70	0.83	0.93	1.48 (1.05–2.10)	0.026	0.039	1.12 (0.80–1.57)	0.500	0.413
rs17694555	22,051,295	0.78	0.83	0.11	1.71 (1.31–2.11)	0.008	0.015	1.79 (0.33–3.25)	0.430	0.386
rs17756311	22,053,895	0.77	0.83	1	1.43 (0.99–2.05)	0.055	0.076	1.05 (0.75–1.49)	0.766	0.665
rs74655961	22054164	0.77	0.83	1	1.46 (1.01–2.10)	0.041	0.058	1.14 (0.82–1.60)	0.430	0.355
rs17694572	22,054,356	0.77	0.83	1	1.46 (1.02–2.10)	0.041	0.058	1.11 (0.80–1.56)	0.528	0.445
rs77728904	22,057,530	—	—	—	1.76 (1.21–2.57)	0.003	0.005	1.16 (0.82–1.64)	0.403	0.281
rs115574830	22,059,061	0.87	1	1	1.70 (1.17–2.48)	0.005	0.007	1.26 (0.90–1.76)	0.186	0.120

AA, African Americans; ASW, AA ancestry; BCP-ALL, B-cell precursor acute lymphoblastic leukaemia; CI, confidence interval; EUR, European ancestry; HA, Hispanic Americans; LD, linkage disequilibrium; MXL, Hispanic ancestry; NA, Native Americans; OR, odds ratio; SNP, single-nucleotide polymorphism.

Highlighted are SNPs with the smallest *P*-values for each stage of the analysis (see Table 1 legend). rs662463 is the only SNP significantly associated with BCP-ALL in AAs (bold).

^*^Correlation (*r*^2^) with rs77728904 in individuals of EUR, MXL and ASW in 1,000 Genomes Phase 1 (ref. 17).

^†^Physical positions on chromosome 9 are from NCBI build 37/hg19.

^‡^HA ancestry defined as >10% NA with % of NA>YRI (African ancestry).

^§^AA ancestry defined as >70% YRI ancestry.

^‖^SNPs in LD (*r*^2^>0.60) with rs77728904 and present in the discovery, replication and multi-ethnic analyses.

^¶^1,000 Genomes population of Mexican ancestry from Los Angeles, CA, used as a proxy for HA ancestry.

^#^1,000 Genomes population of African ancestry in Southwest USA, used as a proxy for AA ancestry.

^**^OR with 95%CI.

^††^Unconditional *P*-values for listed SNPs.

^‡‡^*P*-values for listed SNPs when conditioned on rs3731217.
