# Supplementary material for: A variant at 9p21.3 functionally implicates CDKN2B in paediatric B-cell precursor acute lymphoblastic leukaemia aetiology
Source: Nat Commun. 2016 Feb 12;7:10635. doi: 10.1038/ncomms10635 (PMC4754340; doi:10.1038/ncomms10635)
Supplement: Supplementary Information — Supplementary Figures 1-5, Supplementary Tables 1-7 and Supplementary References. [file ncomms10635-s1.pdf]

Supplementary Figure 1. QC flow chart for the discovery GWAS meta-analysis prior to imputation. The upper boxes contain the initial number of cases/controls, array type, and number of genotyped SNPs prior to QC for each dataset. The middle boxes contain the number of cases/controls and SNPs removed due to QC criteria (in blue italics). The bottom boxes contain the final number of samples and SNPs used for imputation, as well as the number of SNPs in the raw imputation output. QC steps are described in detail in Methods.

Abbreviations: MAF: minor allele frequency; GR: genotyping rate; HWE: Hardy-Weinberg equilibrium; D-M: SNP differential missingness between cases and controls; CR: individual call rate; IC: inbreeding coefficient (|F| > 0.05).

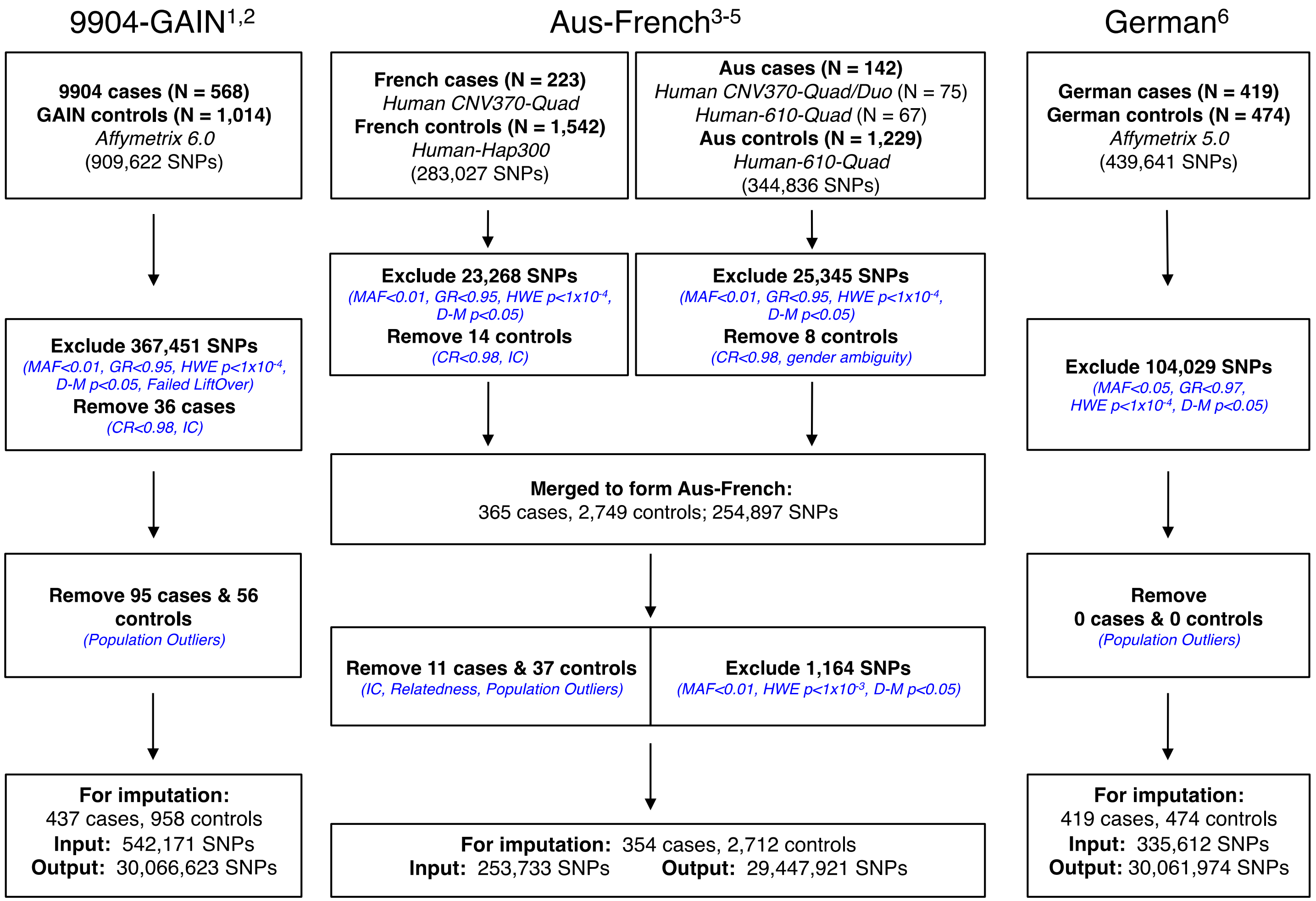

Supplementary Figure 2. QC flow chart for the discovery GWAS meta-analysis after imputation. The upper row of boxes contain the number of SNPs in the raw imputation output and the number removed due to poor imputation (IMPUTE2[7-9] info scores <0.3). The next row of boxes contain the number of remaining SNPs, and the number of SNPs removed by QC following association testing in SNPTEST[10]. The third row of boxes contain the number of SNPs utilized in the meta-analysis (performed with METAL[11]). The lower two rows contain the number of SNPs common to all three discovery sets following the meta-analysis, the number of SNPs removed by post-METAL[11] QC, and the final number of samples and SNPs in the meta-analysis. QC steps are described in detail in Methods.

Abbreviations: MAF: minor allele frequency; β-Info: SNPTEST[10] beta estimate info score; HWE: Hardy-Weinberg equilibrium; NA: SNPTEST[10] unable to fit model to data; I^2: test for heterogeneity[12] among datasets; Indels: insertions or deletions; λ_{GC}: genomic inflation factor lambda[13] calculated using gcontrol2 library in the qqman package[14] in R[15].

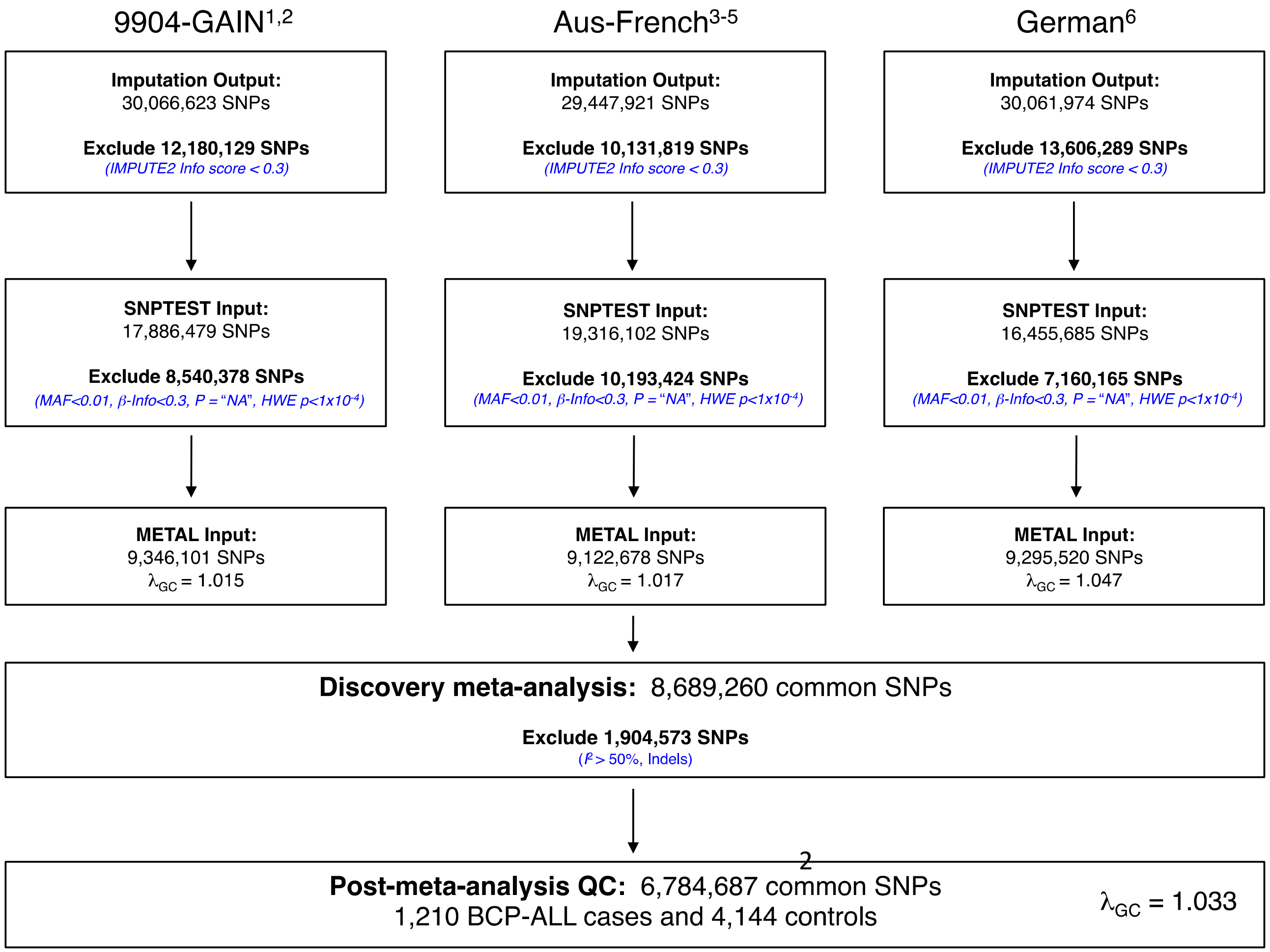

Supplementary Figure 3. **Q-Q plots for the GWAS meta-analysis with (a) all SNPs included and (b) SNPs in loci previously associated with ALL removed (r^2>0.2 with the lead SNP for each locus).** Plots were made using the qqman package[14] in R[15].

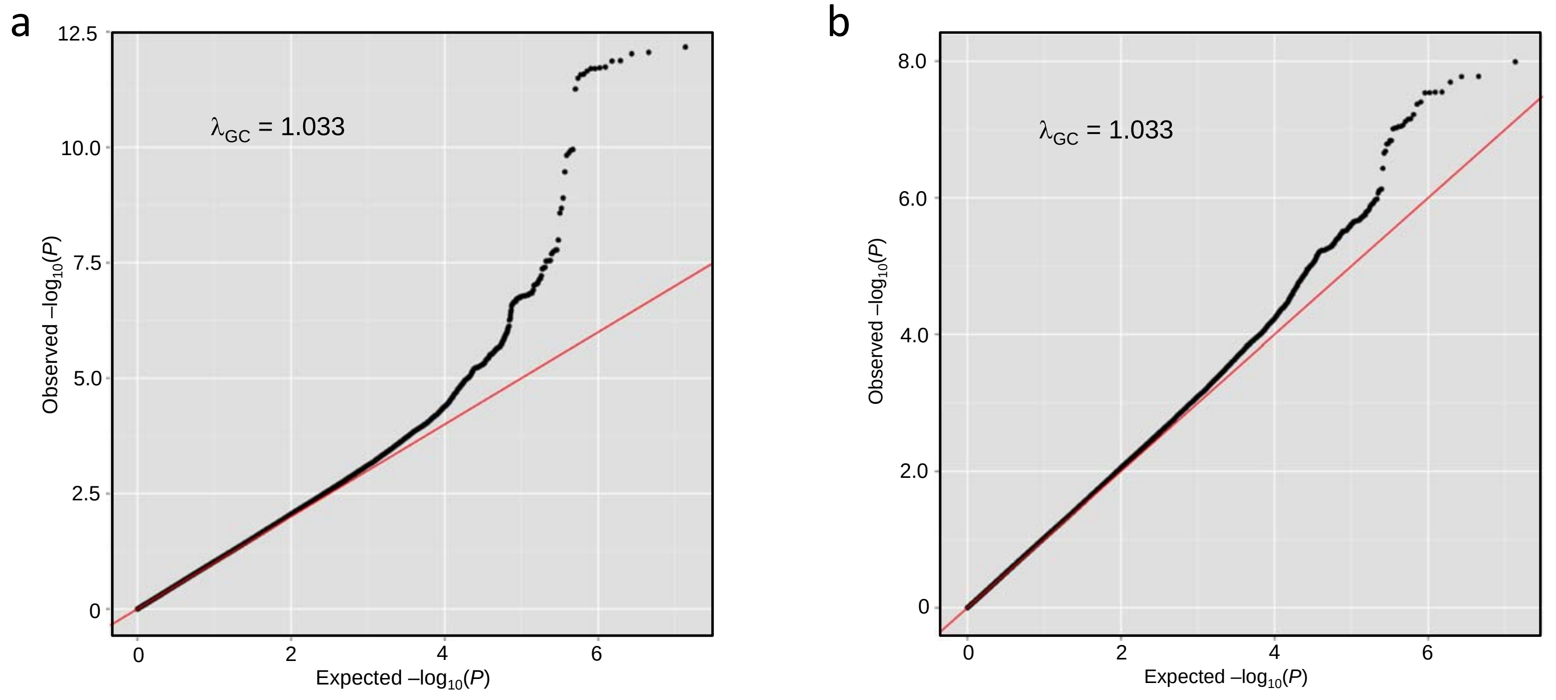

__**Supplementary Figure 4**__**. Correlation analysis of *CDKN2B* expression and the expression of TFs binding to SNPs in the 9p21.3 locus in LCLs from European ancestry individuals[17].** Shown are overall and genotype-dependent co-expression for (**a**) *CDKN2B* and *CEBPB* (shown originally in **Fig. 2**); (**b**) *CDKN2B* and *JUND*; and (**c**) *CDKN2B* and *HOXB9*. *JUND* and *HOXB9* are the only TFs binding to SNPs in the 9p21.3 locus with evidence for genotype-dependent co-expression surpassing a nominal threshold for significance (P_{LRT}<0.05; **Supplementary Table 12**). *JUND* is the only TF with both significant correlation with *CDKN2B* expression and significant genotype-dependent co-expression (**Supplementary Table 12**).

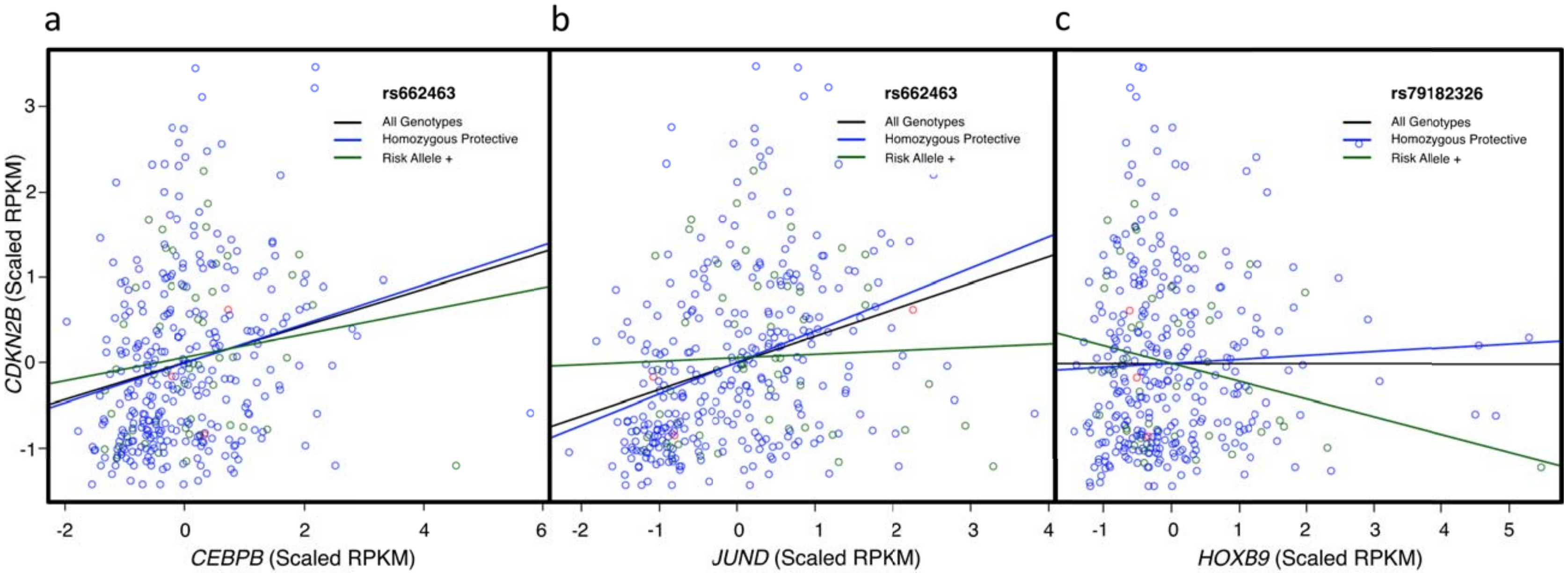

<u>Supplementary Figure 5</u>. Expanded version of Figure 4a showing all protein isoforms encoded by *CDKN2A*. p14^{ARF}, p16□, and p16^{INK4a} are indicated by arrows. The four main exons are labeled, with exon 3 surrounded by a red box. rs3731217 is located in two overlapping intronic splicing elements between exons 1□ and 1□. The figure is adapted from an NCBI AceView plot[18].

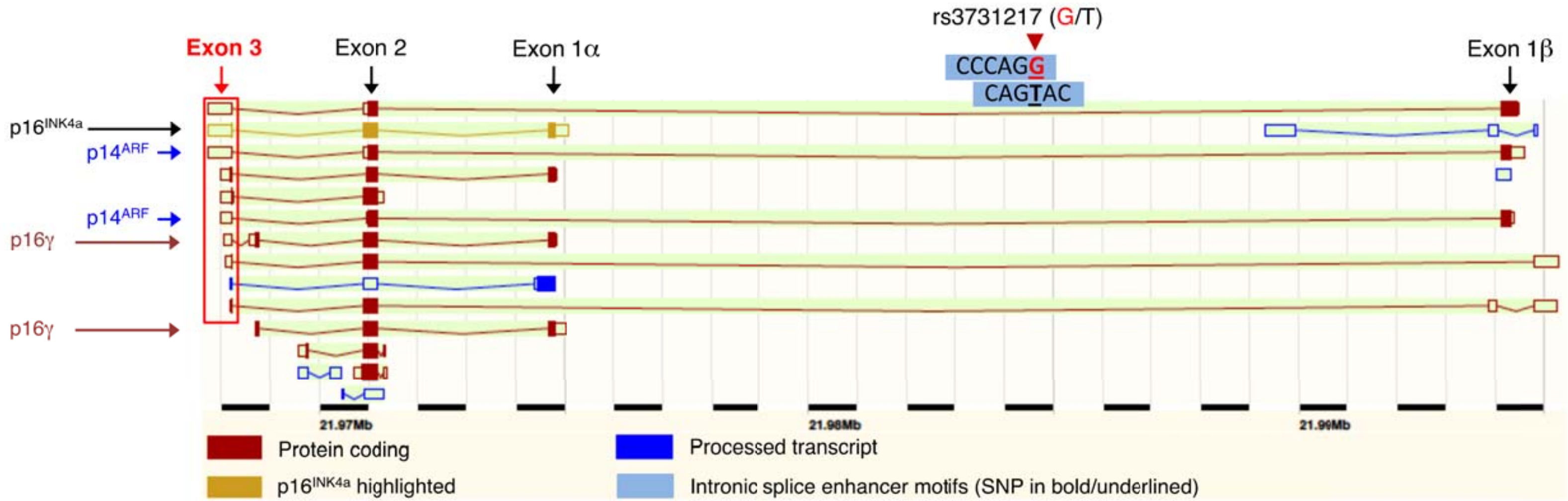

<u>Supplementary Table 1</u>. Association with pediatric BCP-ALL of previously reported BCP-ALL risk variants in the discovery meta-analysis.

SNP	Gene	Chr[a]	I^{2} [b]	$P_{Cochran}$ [c]	OR[d]	OR 95% CI[e]	$P_{discovery}$ [f]
rs7089424	*ARID5B*[19,20]	10	86.7	0.001	1.72	1.55-1.91	8.91×10^{-24}
rs4132601	*IKZF1*[20]	7	65.0	0.057	1.65	1.48-1.84	1.69×10^{-19}
rs2239633	*CEBPE*[20]	14	0.0	0.568	1.26	1.14-1.40	1.12×10^{-5}
rs4266962	*BMI1-PIP42KA*[21]	10	0.0	0.991	1.27	1.13-1.43	8.41×10^{-5}
rs3731217	*CDKN2A*[22]	9	0.0	0.656	0.73	0.62-0.85	5.42×10^{-5}
rs17505102	*TP63*[6]	3	49.4	0.138	1.18	1.02-1.38	0.027
rs3824662	*GATA3*[23,24]	10	0.0	0.659	0.93	0.81-1.06	0.276

[a] Chromosome
[b] Measure of heterogeneity explained due to variance between data sets (%)
[c] *P* value from Cochran's test of heterogeneity between data sets
[d] Odds ratio
[e] 95% confidence interval for odds ratio
[f] *P* values calculated using SNPTEST[10] (see Methods)

Supplementary Table 2. Minor allele frequencies of SNPs in the 9p21.3 locus in the discovery, replication, and multi-ethnic datasets. The number of individuals in each group is listed in parentheses.

		Discovery						Replication[21,25,26]		Multi-ethnic[21,26]			
		9904-GAIN[1,2]		Aus-French[3-5]		German[6]		EA[a]		HA[b]		AA[c]	
SNP	Minor allele	Cases (437)	Controls (958)	Cases (354)	Controls (2,712)	Cases (419)	Controls (474)	Cases (977)	Controls (1,399)	Cases (391)	Controls (1,008)	Cases (203)	Controls (1,363)
rs78545330	A	0.125	0.093	0.151	0.097	0.123	0.091	0.139	0.094	0.096	0.077	0.251	0.220
rs2811712	G	0.131	0.107	0.158	0.104	0.123	0.105	0.148	0.104	0.098	0.080	0.241	0.217
rs3218018	G	0.118	0.085	0.138	0.089	0.116	0.084	0.130	0.087	0.079	0.058	0.086	0.088
rs3218007	T	0.125	0.092	0.150	0.097	0.122	0.091	0.139	0.094	0.095	0.077	0.239	0.215
rs3218005	C	0.125	0.092	0.150	0.097	0.122	0.091	0.139	0.094	0.096	0.077	0.239	0.215
rs3218002	A	0.125	0.092	0.150	0.097	0.122	0.091	0.139	0.094	0.096	0.078	0.239	0.213
rs2069426	T	0.118	0.085	0.138	0.089	0.116	0.084	0.130	0.087	0.079	0.058	0.101	0.088
rs974336	T	0.125	0.092	0.150	0.097	0.122	0.091	0.142	0.094	0.097	0.079	0.251	0.231
rs2069422	G	0.124	0.099	0.145	0.096	0.117	0.099	0.139	0.097	0.083	0.062	0.101	0.082
rs77920300	T	0.124	0.088	0.138	0.089	0.114	0.082	0.131	0.088	0.079	0.058	0.101	0.091
rs116729641	A	0.126	0.087	0.139	0.089	0.114	0.081	0.131	0.088	0.079	0.059	0.101	0.091
rs77284052	T	0.126	0.087	0.138	0.089	0.114	0.081	0.131	0.088	0.079	0.059	0.101	0.091
rs598664	C	0.132	0.102	0.146	0.096	0.115	0.096	0.140	0.098	0.082	0.062	0.101	0.092
rs662463	A	0.133	0.103	0.146	0.097	0.116	0.097	0.140	0.098	0.083	0.065	0.177	0.123
rs79985856	T	0.127	0.087	0.138	0.089	0.114	0.082	0.131	0.088	0.079	0.058	0.101	0.089
rs79182326	T	0.139	0.107	0.154	0.103	0.117	0.101	0.140	0.101	0.082	0.063	0.101	0.088
rs76213463	A	0.127	0.087	0.139	0.089	0.114	0.082	0.130	0.088	0.079	0.058	0.111	0.096
rs80166549	G	0.127	0.087	0.139	0.089	0.114	0.082	0.130	0.088	0.079	0.058	0.111	0.099
rs1333035	G	0.140	0.110	0.160	0.105	0.123	0.104	0.148	0.105	0.097	0.081	0.241	0.223
rs1333034	C	0.133	0.102	0.147	0.097	0.115	0.096	0.140	0.098	0.083	0.061	0.111	0.100
rs17694555	G	0.113	0.082	0.127	0.082	0.104	0.076	0.126	0.084	0.070	0.062	0.005	0.009
rs17756311	A	0.122	0.084	0.126	0.081	0.107	0.078	0.127	0.085	0.074	0.058	0.103	0.098
rs74655961	G	0.123	0.084	0.126	0.080	0.107	0.078	0.126	0.085	0.075	0.057	0.111	0.098
rs17694572	A	0.124	0.085	0.126	0.080	0.109	0.078	0.126	0.085	0.075	0.057	0.108	0.098
rs77728904	C	0.108	0.073	0.119	0.074	0.100	0.068	0.119	0.073	0.075	0.045	0.101	0.088
rs115574830	A	0.116	0.084	0.132	0.087	0.112	0.078	0.125	0.085	0.075	0.047	0.111	0.090

[a] European-American
[b] Hispanic-American

[c] African-American
Highlighted are SNPs with the smallest *P* values for each stage of the analysis (rs77728904 in blue for EA in the discovery, replication, and combined datasets; and rs662463 in green for AA in the multi-ethnic dataset).

<u>Supplementary Table 3</u>. Directly genotyped versus imputed SNPs in the 9p21.3 locus in the discovery datasets. Imputation quality is reported as the IMPUTE2[7-9] info score (higher scores indicate greater imputation certainty).

	9904-GAIN[1,2]		Aus-French[3-5]		German[6]	
SNP	Genotyped?	Info score	Genotyped?	Info score	Genotyped?	Info score
rs78545330	No	0.955	No	0.990	No	0.963
rs2811712	No	0.969	Yes	----	No	0.976
rs3218018	No	0.964	Yes	----	No	0.977
rs3218007	No	0.967	No	0.998	No	0.975
rs3218005	No	0.967	No	0.998	No	0.975
rs3218002	No	0.968	No	0.998	No	0.976
rs2069426	No	0.968	No	1	No	0.980
rs974336	No	0.969	No	0.999	No	0.978
rs2069422	No	0.972	Yes	----	No	0.982
rs77920300	No	0.983	No	0.999	No	0.991
rs116729641	No	0.998	No	0.998	No	0.999
rs77284052	No	0.998	No	0.999	No	0.998
rs598664	No	0.999	No	0.999	No	0.998
rs662463	Yes	----	No	0.997	No	0.996
rs79985856	No	0.999	No	1	No	0.996
rs79182326	No	0.991	No	0.984	No	0.987
rs76213463	No	0.998	No	0.998	No	0.991
rs80166549	No	0.998	No	0.998	No	0.991
rs1333035	No	0.996	No	0.997	No	0.991
rs1333034	No	0.998	Yes	----	No	0.991
rs17694555	No	0.972	No	0.968	No	0.951
rs17756311	Yes	----	No	0.940	Yes	----
rs74655961	No	0.999	No	0.940	No	1
rs17694572	Yes	----	No	0.940	Yes	----
rs77728904	No	0.985	No	0.992	No	0.942
rs115574830	No	0.963	No	0.992	No	0.925

Highlighted are SNPs with the smallest *P* values for each stage of the analysis (rs77728904 in blue for EA in the discovery, replication, and combined datasets; and rs662463 in green for AA in the multi-ethnic dataset).

Supplementary Table 4. Association analysis between BCP-ALL and rs3731217, conditioned on rs77728904 or rs662463. Conditional *P* values are included for each discovery set individually and for the discovery meta-analysis. Results demonstrate that rs3731217 is significantly associated with pediatric BCP-ALL independent of SNPs in the novel 9p21.3 locus.

		Discovery			
SNP	Conditional on	9904-GAIN[*,1,2]	Aus-French[*,3-5]	German[*,6]	Meta[+]
rs3731217	—	1.86×10^{-3}	8.82×10^{-3}	0.441	5.42×10^{-5}
rs3731217	rs77728904	4.62×10^{-3}	0.026	0.646	5.06×10^{-4}
rs3731217	rs662463	4.17×10^{-3}	0.031	0.599	5.16×10^{-4}

Red highlight represents unconditioned rs3731217.
[*]*P* values are calculated in SNPTEST using a frequentist additive missing data likelihood score test that conditions on the expected genotype of the SNP in the "Condition on" column.
[+]*P* values are derived from Z tests calculated using the inverse variance weighting method to combine association evidence across the three discovery datasets.

Supplementary Table 5. Characteristics of BCP-ALL cases in the discovery GWAS and the effect of rs77728904 on each BCP-ALL subtype.

Dataset	Age range (yrs)	Male number	*ETV6-RUNX1*	DT/HHD	Other	Total
9904[1]	1-9	256 (72%)	239 (53%)	227 (50%)	0	452[a]
**OR (SE)**			**1.50 (0.29)**	**1.86 (0.38)**		
Aus-French[3-5]	1-15	180 (51%)	129 (36%)	146 (41%)	81[b] (23%)	354[c]
**OR (SE)**			**1.92 (0.47)**	**1.77 (0.40)**		
German[6]	1-15	229 (55%)	419 (100%)	0	0	419
**OR (SE)**			**1.77 (0.36)**	**1.77 (0.36)**		

[a] Includes 14 cases that have the *ETV6-RUNX1* translocation and are DT/HHD.
[b] Includes 60 hyperdiploid cases of unknown ploidy number (Aus) and 21 hyperdiploid cases with 47-50 chromosomes (French).
[c] Includes 2 cases that have the *ETV6-RUNX1* translocation and are DT/HHD.

Supplementary Table 6. Analysis of power to detect associations between BCP-ALL and SNPs in the rs77728904-tagged locus[a] in AAs.

SNP	MAF in AA[b]	OR[c]	Power in AA[d]	LD (r^2) with rs77728904 EUR[e]	LD (r^2) with rs77728904 ASW[e]
rs78545330	0.303	1.57	0.99	0.77	0.28
rs2811712	0.303	1.52	0.98	0.69	0.28
rs3218018	0.131	1.58	0.92	0.79	0.86
rs3218007	0.303	1.57	0.99	0.77	0.28
rs3218005	0.303	1.57	0.99	0.77	0.28
rs3218002	0.303	1.57	0.99	0.77	0.28
rs2069426	0.131	1.58	0.91	0.79	0.86
rs974336	0.336	1.60	1	0.77	0.30
rs2069422	0.131	1.52	0.86	0.70	0.86
rs77920300	0.123	1.57	0.89	0.79	0.93
rs116729641	0.131	1.57	0.91	0.79	0.86
rs77284052	0.123	1.57	0.89	0.79	0.93
rs598664	0.123	1.51	0.84	0.70	0.93
rs662463	0.180	1.50	0.91	0.70	0.58
rs79985856	0.123	1.56	0.89	0.79	0.93
rs79182326	0.123	1.45	0.77	0.69	0.93
rs76213463	0.123	1.56	0.88	0.79	0.93
rs80166549	0.123	1.56	0.88	0.79	0.93
rs1333035	0.279	1.50	0.97	0.69	0.32
rs1333034	0.123	1.50	0.83	0.70	0.93
rs17694555	0.016	1.59	0.31	0.78	0.11
rs17756311	0.131	1.57	0.91	0.77	1
rs74655961	0.131	1.55	0.89	0.77	1
rs17694572	0.131	1.54	0.89	0.77	1
rs77728904	0.131	1.74	0.98	----	----
rs115574830	0.131	1.55	0.89	0.87	1

[a] SNPs in r^2_{EUR}>0.60 with rs77728904 and present in discovery, replication, and multi-ethnic analyses.
[b] MAF is the minor allele frequency estimated from 1000 Genomes[27] ASW population.
[c] OR is the odds ratio of the risk allele measured in the EA meta-analysis.
[d] Power calculated using the MAF and OR listed, assuming a disease prevalence of 1% and an additive genetic model.
[e] r^2 measured using 1000 Genomes[27] data from EUR and ASW.

<u>Supplementary Table 7</u>. SNPs in the rs77728904-tagged locus are c*is*-Expression Quantitative Trait Loci for *CDKN2B* in whole blood using data from the GTEx project[28] (n=168 samples).

SNP[a]	$P_{additive}$ Value[b]	Effect Size[c]
**rs77728904**	$6.1\text{x}10^{-7}$	0.51
**rs1333035**	$6.2\text{x}10^{-7}$	0.43
**rs3218005**	$7.3\text{x}10^{-7}$	0.43
**rs3218007**	$7.6\text{x}10^{-7}$	0.43
**rs662463**	$8.7\text{x}10^{-7}$	0.44
**rs1333034**	$9.3\text{x}10^{-7}$	0.45
**rs76213463**	$9.3\text{x}10^{-7}$	0.45
**rs80166549**	$9.3\text{x}10^{-7}$	0.45
rs2069422[d]	$1.0\text{x}10^{-6}$	0.46
**rs2069426**	$1.0\text{x}10^{-6}$	0.46
**rs116729641**	$1.0\text{x}10^{-6}$	0.45
**rs3218002**	$1.0\text{x}10^{-6}$	0.42
**rs78545330**	$1.1\text{x}10^{-6}$	0.43
**rs598664**	$1.2\text{x}10^{-6}$	0.45
**rs77920300**	$1.2\text{x}10^{-6}$	0.46
**rs77284052**	$1.3\text{x}10^{-6}$	0.45
**rs2811712**	$1.3\text{x}10^{-6}$	0.42
**rs79182326**	$1.9\text{x}10^{-6}$	0.45
**rs79985856**	$1.9\text{x}10^{-6}$	0.45
**rs3218018**	$1.9\text{x}10^{-6}$	0.45
**rs115574830**	$2.1\text{x}10^{-6}$	0.46
rs17694555	NA	NA
rs17756311	NA	NA
rs17694572	NA	NA
rs74655961	NA	NA
rs974336	NA	NA

[a] SNPs in bold have r^2>0.60 with rs77728904 and discovery association $P<10^{-5}$.
[b] SNPs listed in order from most to least significant *P* value (calculated in Matrix eQTL using a t-test).
[c] Effect size is reported as standard deviations of a standard Normal distribution with respect to minor allele.
[d] SNP has r^2>0.60 with rs77728904 but has discovery association $P_{discovery}>10^{-5}$.
NA, No eQTL information available for SNP in online GTEx portal[28] (http://www.gtexportal.org/home/).

Supplementary References

1. Borowitz, M.J. *et al.* Clinical significance of minimal residual disease in childhood acute lymphoblastic leukemia and its relationship to other prognostic factors: a Children's Oncology Group study. *Blood* **111**, 5477-85 (2008).
2. Manolio, T.A. *et al.* New models of collaboration in genome-wide association studies: the Genetic Association Information Network. *Nat Genet* **39**, 1045-51 (2007).
3. Orsi, L. *et al.* Genetic polymorphisms and childhood acute lymphoblastic leukemia: GWAS of the ESCALE study (SFCE). *Leukemia* **26**, 2561-4 (2012).
4. Rudant, J. *et al.* Childhood acute leukemia, early common infections, and allergy: The ESCALE Study. *Am J Epidemiol* **172**, 1015-27 (2010).
5. Evans, T.J. et al. Confirmation of Childhood Acute Lymphoblastic Leukemia Variants, *ARID5B* and *IKZF1*, and Interaction with Parental Environmental Exposures. PLoS One 9, e110255 (2014).
6. Ellinghaus, E. *et al.* Identification of germline susceptibility loci in ETV6-RUNX1-rearranged childhood acute lymphoblastic leukemia. *Leukemia* **26**, 902-9 (2012).
7. Howie, B.N., Donnelly, P., & Marchini, J. A Flexible and Accurate Genotype Imputation Method for the Next Generation of Genome-Wide Association Studies. *PLoS Genet.* **5**, e1000529 (2009).
8. Howie, B.N., Marchini, J., & Stephens, M. Genotype imputation with thousands of genomes. *G3: Genes, Genomics, Genetics* **1**, 457-470 (2011).
9. Howie, B., Fuchsberger, C., Stephens, M., Marchini, J. & Abecasis, G.R. Fast and accurate genotype imputation in genome-wide association studies through pre-phasing. *Nat Genet* **44**, 955-9 (2012).
10. Marchini, J., Howie, B., Myers, S., McVean, G. & Donnelly, P. A new multipoint method for genome-wide association studies by imputation of genotypes. *Nat Genet* **39**, 906-13 (2007).
11. Willer, C.J., Li, Y. & Abecasis, G.R. METAL: fast and efficient meta-analysis of genomewide association scans. *Bioinformatics* **26**, 2190-1 (2010).
12. Higgins, J.P., Thompson, S.G., Deeks, J.J. & Altman, D.G. Measuring inconsistency in meta-analyses. *BMJ* **327**, 557-60 (2003).
13. Devlin, B. & Roeder, K. Genomic control for association studies. *Biometrics* **55**, 997-1004 (1999).
14. Turner, S.D. qqman: an R package for visualizing GWAS results using Q-Q and manhattan plots. *biorXiv* DOI: 10.1101/005165.
15. R Development Core Team (2008). R: A language and environment for statistical computing. R Foundation for Statistical Computing, Vienna, Austria. ISBN 3-900051-07-0 (http://www.R-project.org).
16. Pruim, R.J. *et al.* LocusZoom: regional visualization of genome-wide association scan results. *Bioinformatics* **26**, 2336-7 (2010).
17. Lappalainen, T. *et al.* Transcriptome and genome sequencing uncovers functional variation in humans. *Nature* **501**, 506-11 (2013).
18. Thierry-Mieg, D. & Thierry-Mieg, J. AceView: a comprehensive cDNA-supported gene and transcripts annotation. *Genome Biol.* **7**, S12.1-S12.14 (2006).
19. Trevino, L.R. *et al.* Germline genomic variants associated with childhood acute lymphoblastic leukemia. *Nat Genet* **41**, 1001-5 (2009).

20. Papaemmanuil, E. *et al.* Loci on 7p12.2, 10q21.2 and 14q11.2 are associated with risk of childhood acute lymphoblastic leukemia. *Nat Genet* **41**, 1006-10 (2009).
21. Xu, H. *et al.* Novel susceptibility variants at 10p12.31-12.2 for childhood acute lymphoblastic leukemia in ethnically diverse populations. *J Natl Cancer Inst* **105**, 733-42 (2013).
22. Sherborne, A.L. *et al.* Variation in CDKN2A at 9p21.3 influences childhood acute lymphoblastic leukemia risk. *Nat Genet* **42**, 492-4 (2010).
23. Migliorini, G. *et al.* Variation at 10p12.2 and 10p14 influences risk of childhood B-cell acute lymphoblastic leukemia and phenotype. *Blood* **122**, 3298-307 (2013).
24. Perez-Andreu, V. *et al*. Inherited *GATA3* variants are associated with Ph-like childhood acute lymphoblastic leukemia and risk of relapse. *Nat Genet* **45**, 1494-98 (2013).
25. Walsh, K.M. *et al.* Novel childhood ALL susceptibility locus BMI1-PIP4K2A is specifically associated with the hyperdiploid subtype. *Blood* **121**, 4808-9 (2013).
26. Bild, D.E. *et al.* Multi-ethnic study of atherosclerosis: objectives and design. *Am J Epidemiol* **156**, 871-81 (2002).
27. McVean, G.A. *et al*. An integrated map of genetic variation from 1,092 human genomes. *Nature* **491**, 56-65 (2012).
28. Lonsdale, J. *et al.* The Genotype-Tissue Expression (GTEx) project. *Nat Genet* **45**, 580-5 (2013).
29. Ward, L.D. & Kellis, M. HaploReg: a resource for exploring chromatin states, conservation, and regulatory motif alterations within sets of genetically linked variants. *Nucleic Acids Res*. **40**, D930-934 (2012).
30. Dunham, I. *et al.* An integrated encyclopedia of DNA elements in the human genome. *Nature* **489**, 57-74 (2012).
31. Matys, V. *et al*. TRANSFAC and its module TRANSCompel: transcriptional gene regulation in eukaryotes. *Nucleic Acids Res*. **34**, D108-110 (2006).
32. Sandelin, A., Alkema, W., Engstrom, P., Wasserman, W.W. & Lenhard, B. JASPAR: an open-access database for eukaryotic transcription factor binding profiles. *Nucleic Acids Res*. **32**, D91-D94 (2004).
33. Berger, M.F. *et al*. Compact, universal DNA microarrays to comprehensively determine transcription-factor binding site specificities. *Nat. Biotechnol*. **24**, 1429-1435 (2006).
34. Berger, M.F. *et al*. Variation in homeodomain DNA binding revealed by high-resolution analysis of sequence preferences. *Cell* **133**, 1266-1276 (2008).
35. Badis, G. *et al*. Diversity and complexity in DNA recognition by transcription factors. *Science* **324**, 1720-1723 (2009).
36. Bernstein, B.E. *et al*. The NIH Roadmap Epigenomics Mapping Consortium. *Nat. Biotechnol*. **28**, 1045-1048 (2010).
37. Boyle, A.P. *et al*. Annotation of functional variation in personal genomes using RegulomeDB. *Genome Res*. **22**,1790-1797 (2012).
38. Stranger, B.E. *et al.* Patterns of cis regulatory variation in diverse human populations. *PLoS Genet* **8**, e1002639 (2012).
39. The International HapMap 3 Consortium. Integrating common and rare genetic variation in diverse human populations. *Nature* **467**, 52-58 (2010).
